# Deep learning in image-based breast and cervical cancer detection: a systematic review and meta-analysis

**DOI:** 10.1038/s41746-022-00559-z

**Published:** 2022-02-15

**Authors:** Peng Xue, Jiaxu Wang, Dongxu Qin, Huijiao Yan, Yimin Qu, Samuel Seery, Yu Jiang, Youlin Qiao

**Affiliations:** 1 Department of Epidemiology and Biostatistics, School of Population Medicine and Public Health, Chinese Academy of Medical Sciences and Peking Union Medical College, Beijing, 100730 China; 2 National Cancer Center/National Clinical Research Center for Cancer/Cancer Hospital, Chinese Academy of Medical Sciences and Peking Union Medical College, Beijing, 100021 China; 3 School of Humanities and Social Sciences, Chinese Academy of Medical Sciences and Peking Union Medical College, Beijing, 100730 China; 4 Faculty of Health and Medicine, Division of Health Research, Lancaster University, Lancaster, LA1 4YW United Kingdom

**Keywords:** Cancer prevention, Diagnosis

## Abstract

Accurate early detection of breast and cervical cancer is vital for treatment success. Here, we conduct a meta-analysis to assess the diagnostic performance of deep learning (DL) algorithms for early breast and cervical cancer identification. Four subgroups are also investigated: cancer type (breast or cervical), validation type (internal or external), imaging modalities (mammography, ultrasound, cytology, or colposcopy), and DL algorithms versus clinicians. Thirty-five studies are deemed eligible for systematic review, 20 of which are meta-analyzed, with a pooled sensitivity of 88% (95% CI 85–90%), specificity of 84% (79–87%), and AUC of 0.92 (0.90–0.94). Acceptable diagnostic performance with analogous DL algorithms was highlighted across all subgroups. Therefore, DL algorithms could be useful for detecting breast and cervical cancer using medical imaging, having equivalent performance to human clinicians. However, this tentative assertion is based on studies with relatively poor designs and reporting, which likely caused bias and overestimated algorithm performance. Evidence-based, standardized guidelines around study methods and reporting are required to improve the quality of DL research.

## Introduction

Female breast and cervical cancer remain as major contributors to the burden of cancer^1,2^. The World Health Organization (WHO) reported that approximately 2.86 million new cases (14.8% of all cancer cases) and 1.03 million deaths (10.3% of all cancer deaths) were recorded worldwide in 2020^3^. This disproportionately affects women, especially in low- and middle-income countries (LMICs), which can be largely attributed to more advanced stage diagnoses, limited access to early diagnostics, and suboptimal treatment^4,5^. Population-based cancer screening in high-income countries might not be as effective in LMICs, due to limited resources for treatment and palliative care^6,7^. Integrative screening for cancer is a complex procedure that needs to take biological and social determinants, as well as ethical constraints into consideration, and as is already known, early detection of breast and cervical cancers are associated with improved prognosis and survival^8,9^. Therefore, it is vital to select the most accurate and reliable technologies that are capable of identifying early symptoms.

Medical imaging plays an essential role in tumor detection, especially within progressively digitized cancer care services. For example, mammography and ultrasound, as well as cytology and colposcopy are commonly used in clinical practice^10–14^. However, fragmented health systems in LMICs may lack infrastructure and perhaps the manpower required to ensure high-quality screening, diagnosis, and treatment. This hinders the universality of traditional detection technologies mentioned above, which require sophisticated training^15^. Furthermore, there may be substantial inter- and intraoperator variability which affects both machine and human performances. Therefore, the interpretation of medical imaging is vulnerable to human error. Of course, experienced doctors tend to be more accurate although their expertise is not always readily available for marginalized populations, or for those living in remote areas. Resource-based testing and deployment of effective interventions together could reduce cancer morbidity and mortality in LMICs^16^. In line with this, an ideal detection technology for LMICs should at least have low training needs.

Deep learning (DL), as a subset of artificial intelligence (AI), could be applied to medical imaging and has shown promise in automatic detection^17,18^. While media headlines tend to overemphasize the polarization of DL model findings^19^, few have demonstrated inferiority or superiority. However, the Food and Drug Administration (FDA) has approved a select number of DL-based diagnosis tools for clinical practice, even though further critical appraisal and independent quality assessments are pending^20,21^. To date, there are few medical imaging specialty-specific systematic reviews such as this, which assess the diagnostic performance of DL algorithms, particularly in breast and cervical cancer.

## Results

### Study selection and characteristics

**Fig. 1:**
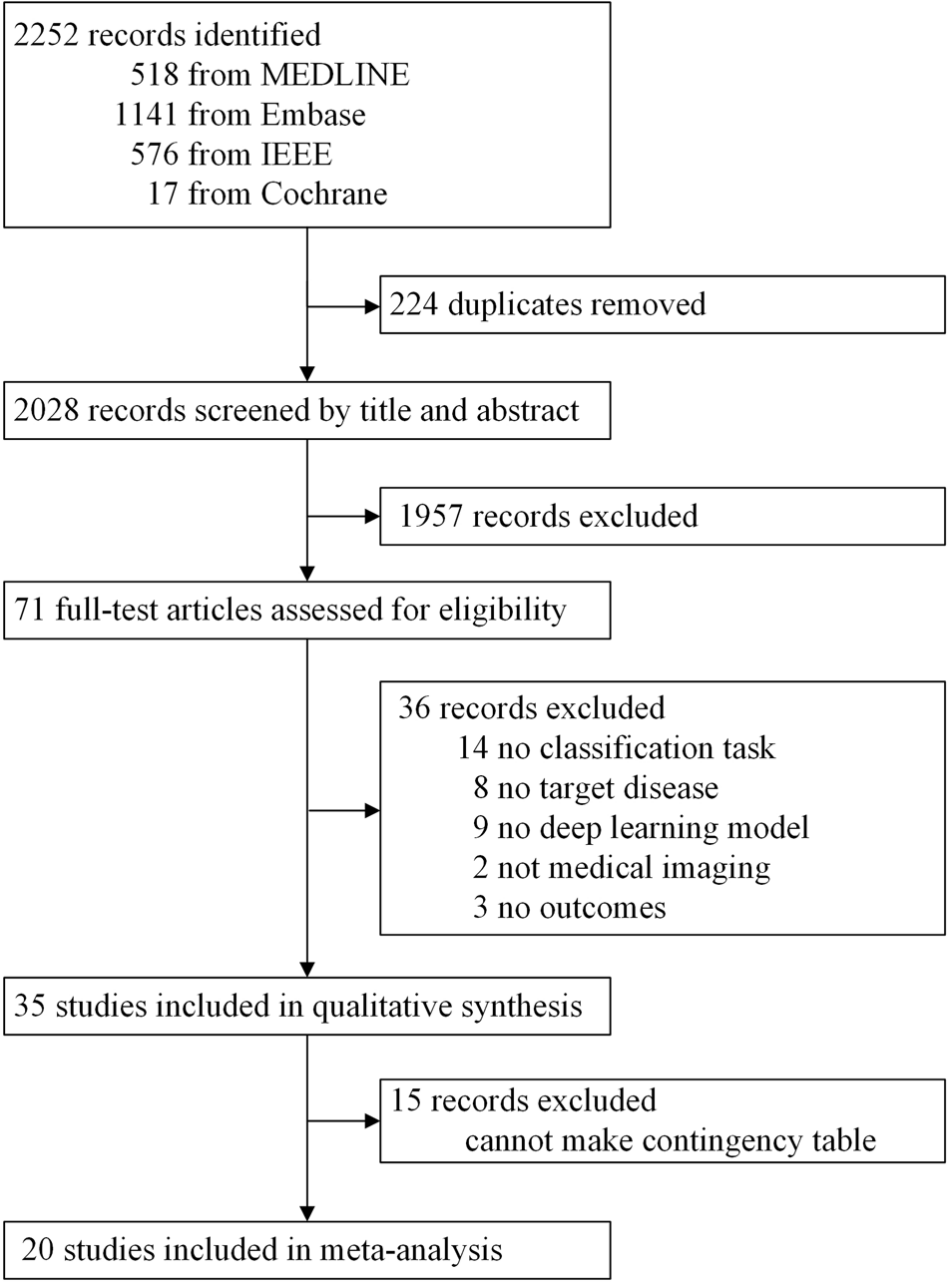
PRISMA flowchart of study selection. Displayed is the PRISMA (preferred reporting items for systematic reviews and meta-analyses) flow of search methodology and literature selection process.

Our search initially identified 2252 records, of which 2028 were screened after removing 224 duplicates. 1957 were also excluded as they did not fulfil our predetermined inclusion criteria. We assessed 71 full-text articles and a further 36 articles were excluded. 25 of these articles focused on breast cancer, and 10 were on cervical cancer (see Fig. 1). Study characteristics are summarized in Tables 1–3.

**Table 1:** Study design and basic demographics.

First author and year	Participants			
	Inclusion criteria	Exclusion criteria	*N*	Mean or median age (SD; range)
Xiao et al.^49^ *	Had breast lesions clearly visualized by ultrasound; Underwent biopsy and had pathological results; provided informed consent.	Patients who were pregnant or lactating; patients who had breast biopsy or were undergoing neoadjuvant chemotherapy or radiotherapy.	389	46.86 (13.03; 19–84)
Zhang et al.^50^ *	NR	Pathological results were neither benign nor malignant; Patients with BI-RADS 1 or 2 and abnormal mammography results; patients who were diagnosed with Paget’s disease but had no masses in the breasts.	2062	NR
Zhou et al.^51^ *	Images were scanned under the same MR protocol; The lesion had complete pathology results; Imaging reports had definite BI-RADS category diagnosed; Lesions were a) solitary in one breast or b) in both breasts with the same BI-RADS and pathological results.	Normal or typical background parenchyma enhancement in bilateral breasts was eliminated.	1537	47.5 (11.8; NR)
Agnes et al.^52^	NR	NR	NR	NR
Tanaka et al.^53^ *	women with breast masses who were referred for further examination after their initial screening examination of breast cancer and then underwent ultrasonography and pathological examination.	Typical cysts; mass lesions ≥ 4.5 cm diameter	NR	NR
Becker et al.^35^	Patients with postsurgical scars, initially indeterminate, or malignant lesions with histological diagnoses or 2 years follow up.	Patients with normal breast ultrasound, and all patients with lesions classified as clearly benign, except for patients with prior breast-conserving surgical treatment.	632	53 (15; 15–91)
Kyono et al.^54^	Women recalled after routine breast screening between ages of 47–73 or women with a family history of breast cancer attending annual screening between ages of 40–49.	NR	2000	NR (NR; 47–73)
Qi et al.^55^ *	NR	NR	2047	NR
Salim et al.^56^ *	Women aged 40–74 years who were diagnosed as having breast cancer, who had a complete screening examination prior to diagnosis, had no prior breast cancer, did not have implants.	With a cancer diagnosis that had ≥ 12 months between the examination and diagnosis date.	8805	54.5 (16.1; 40–74)
Zhang et al.^57^	NR	NR	121	NR
Wang et al.^58^	NR	NR	263	51.4 (9.8; 28–76)
Li et al.^59^	NR	NR	124	NR
Mckinney et al.^60^	NR	Cases without follow-up were excluded from the test set.	28953	NR
Shen et al.^61^	NR	NR	1249	NR
Suh et al.^62^ *	18 years or older and not having a history of previous breast surgery.	Subjects without medical records or pathological confirmation for a suspicious breast lesion, missing mammograms, or having poor-quality mammograms.	1501	48.9 (11.1; NR)
O’Connell et al.^63^	Adult females or males recommended for ultrasound-guided breast lesion biopsy or ultrasound follow-up with at least one suspicious lesion; age ≥ 18 years.	Unable to read and understand English at the University of Rochester; patients with diagnosis of breast cancer in the same quadrant; unwilling to undergo study procedures and informed consent.	299	52.3 (NR; NR)
Ruiz et al.^64^	Women presenting for screening with no symptoms or concerns.	Women with implants and/or a history of breast cancer.	240	62 (53–66; 39–89)
Adachi et al.^65^ *	Patients who underwent DCE breast MRI; patients who were diagnosed with benign or malignant lesions by pathology or a follow-up examination at more than one year.	Patients who were treated with breast surgery, hormonal therapy, chemotherapy, or radiation therapy; age ≤ 20 years.	371	NR
Samala et al.^66^	NR	NR	2242	51.7 (NR; 24–82)
Schaffter et al.^67^	NR	NR	153588	56.1 (NR; NR)
Kim et al.^68^ *	NR	NR	172230	50.3 (10; NR)
Wang et al.^69^	All nodules of patients were newly discovered and untreated; patients had undertaken ABUS scan; definite pathological benign and malignant; the image quality of ABUS examination was good enough to show the entire margin of the lesion, no matter distinct or indistinct.	Non-nodular breast disease; ABUS artifact was obvious and the poor images quality; ABUS was not available; patients received chemotherapy, radiation therapy or surgical local resection before ABUS scan.	264	54.31 (9.68; 37–75)
Yu et al.^70^	Pathological results clearly; at least 2D mode US images available, but preferably CDFI and PW mode images. Without blurred images or color overflow.	A foreign-body in the breast; other metastatic tumors or co-infection with HIV; measurement markers, arrows, or puncture needles within the image;	3623	42.5 (NR; 11–95)
Sasaki et al.^71^ *	Patients undergone bilateral mammography; patients in whom ultrasonography had established the presence or absence of a lesion; patients in whom a lesion, if present, had been diagnosed as being benign or malignant by cytology or histology; normal patients in whom ultrasonography had revealed no lesion and who had been followed up for at least 1 year.	NR	310	50 (NR; 20–93)
Zhang et al.^72^	NR	NR	2620	NR
Bao et al.^73^ *	Aged 20–65 years participated in the program.	NR	703103	NR (NR; 20–65)
Holmström et al.^74^ *	Nonpregnant aged between 18-64 years, confirmed HIV positivity, and signed informed consent.	NR	740	41.8 (10.3; 18–64)
Cho et al.^75^ *	Age ≥18 years, not pregnant, had no history of cervical surgery, and had Pap test results. All lesions were pathologically confirmed by conization biopsy, and normal were defined as those with normal Pap test results.	NR	791	NR (NR; 18–94)
Bao et al.^76^ *	Aged 25–64 years; samples were processed with liquid-based method. done with HPV testing, and diagnosed by colposcopy-directed biopsy.	NR	2145	38.4 (6.7; 25–46)
Hu et al.^77^ *	NR	No image, multiple colpo sessions, inadequate histology.	9406	35 (NR;18–94)
Hunt et al.^78^ *	Abnormal cervical screening test, age ≥18 years, intact uterine cervix, not pregnant, no known allergy to the fluorescent dye used for HRME imaging, does not belong to an indigenous Brazilian population;	unable to provide informed consent; prior treatment history; pregnant; other clinical considerations.	1486	40 (12.1; NR)
Wentzensen et al.^79^ *	Women aged ≥18 years referred to colposcopy.	NR	4253	NR
Xue et al.^39^ *	Aged 24-65 years with indications for the need for colposcopy imaging and biopsy, and those who were pathologically confirmed.	Empty or invalid images, low quality, unsatisfactory images, information loss.	19435	NR (NR; 24–65)
Yu et al.^80^ *	NR	NR	679	NR
Yuan et al.^40^ *	NR	Without complete clinical and pathological information; without biopsies; pathologically diagnosed as invasive cervical cancer or glandular intraepithelial lesions; poor-quality colposcopy images.	22330	NR (NR; 20–66)

*DCE* dynamic contrast enhanced, *NR* not reported, *MRI* magnetic resonance imaging, *BI-RADS* breast imaging reporting and data system, *MR* magnetic resonance, *ABUS* automated breast ultrasound, *CDFI* color doppler flow imaging, *PW* pulsed wave, *HIV* human immunodeficiency virus, *HRME* high-resolution microendoscopy, *DS* dual stained.

*20 studies included in the meta-analysis.

**Table 2:** Methods of model training and validation.

First author and year	Focus	Reference standard	Type of internal validation	External validation	DL versus clinician
Xiao et al.^49^ *	Breast cancer	Histopathology	NR	Yes	Yes
Zhang et al.^50^ *	Breast cancer	Histopathology, immunohistochemistry	Random split-sample validation	Yes	No
Zhou et al.^51^ *	Breast cancer	Histopathology, expert consensus	Random split-sample validation	No	Yes
Agnes et al.^52^	Breast cancer	Histopathology	NR	No	No
Tanaka et al.^53^ *	Breast cancer	Histopathology, two-year follow-up	Random split-sample validation	No	No
Becker et al.^35^	Breast cancer	Histopathology, two-year follow-up	Random split-sample validation	No	No
Kyono et al.^54^	Breast cancer	Histopathology, follow-up, expert consensus	Ten-fold cross validation	No	No
Qi et al.^55^ *	Breast cancer	Histopathology	Random split-sample validation	No	No
Salim et al.^56^ *	Breast cancer	Histopathology, two-year follow-up	NR	Yes	Yes
Zhang et al.^57^	Breast cancer	Histopathology	NR	No	No
Wang et al.^58^	Breast cancer	Histopathology, two-year follow-up	Five-fold cross validation	No	No
Li et al.^59^	Breast cancer	Histopathology	Five-fold cross validation	No	No
Mckinney et al.^60^	Breast cancer	Histopathology, multiple years of follow-up	NR	Yes	No
Shen et al.^61^	Breast cancer	Histopathology	Random split-sample validation	No	No
Suh et al.^62^ *	Breast cancer	Histopathology	Random split-sample validation	No	No
O’Connell et al.^63^	Breast cancer	Histopathology, two-year follow-up	NR	Yes	No
Ruiz et al.^64^	Breast cancer	Histopathology, one-year follow-up	NR	Yes	Yes
Adachi et al.^65^ *	Breast cancer	Histopathology, at least one-year follow-up	Random split-sample validation	No	Yes
Samala et al.^66^	Breast cancer	Histopathology	N-fold cross validation	No	No
Schaffter et al.^67^	Breast cancer	Histopathology, follow-up	Random split-sample validation	Yes	No
Kim et al.^68^ *	Breast cancer	Histopathology, at least one-year follow-up	Random split-sample validation	Yes	Yes
Wang et al.^69^	Breast cancer	Histopathology	Random split-sample validation	No	No
Yu et al.^70^	Breast cancer	Histopathology	Random split-sample validation	No	No
Sasaki et al.^71^ *	Breast cancer	Histopathology, cytology, at least one-year follow-up	NR	Yes	Yes
Zhang et al.^72^	Breast cancer	Histopathology	NR	No	No
Heling Bao et al.^73^ *	Cervical cancer	Histopathology	NR	Yes	Yes
Holmström et al.^74^ *	Cervical cancer	Histopathology	NR	No	Yes
Cho et al.^75^ *	Cervical cancer	Histopathology	Random split-sample validation	No	No
Bao et al.^76^ *	Cervical cancer	Histopathology	NR	Yes	No
Hu et al.^77^ *	Cervical cancer	Histopathology	Random split-sample validation	No	No
Hunt et al.^78^ *	Cervical cancer	Histopathology	Random split-sample validation	No	Yes
Wentzensen et al.^79^ *	Cervical cancer	Histopathology	Random split-sample validation	No	Yes
Xue et al.^39^ *	Cervical cancer	Histopathology	Random split-sample validation	No	Yes
Yu et al.^80^ *	Cervical cancer	Histopathology	Random split-sample validation	No	No
Yuan et al.^40^ *	Cervical cancer	Histopathology	Random split-sample validation	No	No

*NR* not reported, *DL* deep learning. *20 studies included in the meta-analysis.

*20 studies included in the meta-analysis.

**Table 3:** Indicators, algorithms and data sources.

First author and year	Indicator definition			Algorithm		Data source			
	Device	Exclusion of poor-quality imaging	Heatmap provided	Algorithm architecture	Transfer learning applied	Source of data	Number of images for training/internal/external	Data range	Open access data
Xiao et al.^49^ *	Ultrasound	NR	No	CNN	No	Prospective study, data from Peking Union Medical College Hospital.	NR/NR/451	2018.01–2018.12	No
Zhang et al.^50^ *	Ultrasound	Yes	No	CNN	Yes	Retrospective study, training data from Harbin Medical University Cancer Hospital; external data from the First Affiliated Hospital of Harbin Medical University.	2822/447/210	NR	No
Zhou et al.^51^ *	MRI	Yes	Yes	DenseNet	No	Retrospective study, data from Chinese University of Hong Kong.	1230/307/NR	2013.03–2016.12	No
Agnes et al.^52^	Mammography	NR	No	MA-CNN	No	Retrospective study, data from mini-Mammographic Image Analysis Society database.	322/NR/NR	NR	Yes
Tanaka et al.^53^ *	Ultrasound	NR	Yes	VGG19, ResNet152	Yes	Retrospective study, data from Japan Association of Breast Thyroid Sonology.	1382/154/NR	2011.11–2015.12	No
Becker et al.^35^	Ultrasound	NR	Yes	CNN	No	Retrospective study, data from university hospital of Zurich, Switzerland.	445/192/NR	2014.01–2014.12	No
Kyono et al.^54^	Mammography	NR	No	CNN	No	Retrospective study, data from UK National Health Service Breast Screening Program Centers.	1800/200/NR	NR	No
Qi et al.^55^ *	Ultrasound	NR	Yes	GoogLeNet	Yes	Retrospective study, data from West China Hospital, Sichuan University.	6786/1359/NR	2014.10–2017.08	No
Salim et al.^56^ *	Mammography	NR	No	ResNet-34, MobileNet	No	Retrospective study, data from secondary analysis of a population-based mammography screening cohort in Swedish Cohort of Screen-Age Women.	NR/NR/113663	2008–2015	No
Zhang et al.^57^	Ultrasound	NR	No	Deep polynomial networks	No	Retrospective study, data source is not clear.	NR/NR/NR	NR	No
Wang et al.^58^	Ultrasound	NR	No	Inception-v3 CNN	No	Retrospective study, data from Jeonbuk National University Hospital.	252/64/NR	2012.03–2018.03	No
Li et al.^59^	Ultrasound	NR	No	YOLO-v3	No	Retrospective study, data from Peking University People’s Hospital.	3124/10812/NR	2018.10–2019.03	No
Mckinney et al.^60^	Mammography	NR	No	CNN	No	Retrospective study, data 1 from two screening centers in England, data 2 from one medical center in USA.	25856/NR/3097	2001–2018	No
Shen et al.^61^	Mammography	NR	Yes	VGG, Resnet	Yes	Retrospective study, data from CBIS-DDSM website.	2102/376/NR	NR	Yes
Suh et al.^62^ *	Mammography	Yes	Yes	DenseNet-169, EfficientNet-B5	No	Retrospective study, data from Hallym University Sacred Heart Hospital.	2701/301/NR	2007.02–2015.05	No
O’Connell et al.^63^	Ultrasound	NR	No	CNN	No	Prospective study, data from University of Rochester and University Hospital Palermo, Italy.	NR/NR/299	2018–2019	No
Ruiz et al.^64^	Mammography	Yes	No	CNN	No	Retrospective study, data from two institutes in the US and Europe.	NR/NR/240	2013–2017	No
Adachi et al.^65^ *	MRI	NR	No	RetinaNet	No	Retrospective study, data from Tokyo Medical and Dental University hospital.	286/85/NR	2014.03–2018.10	No
Samala et al.^66^	Mammography	NR	No	ImageNet DCNN	Yes	Retrospective study, data from University of Michigan Health System and the Digital Database for Screening Mammography.	1335/907/NR	2001–2006	No
Schaffter et al.^67^	Mammography	NR	No	Faster-RCNN	No	Retrospective study, data from Kaiser Permanente Washington and Karolinska Institute.	100974/43257/166578	2016.09–2017.11	No
Kim et al.^68^ *	Mammography	NR	Yes	ResNet-34	No	Retrospective study, data from five institutions in South Korea, USA.	166968/3262/320	2000.01–2018.12	No
Wang et al.^69^	Ultrasound	Yes	No	3D U-Net	No	Retrospective study, data from the First Affiliated Hospital of Xi’an Jiao tong University.	254/73/NR	2018.06–2019.05	No
Yu et al.^70^	Ultrasound	Yes	No	ResNet50, FPN	No	Retrospective study, data from 13 Chinese hospitals.	7835/7813/NR	2016.01–2019.12	No
Sasaki et al.^71^ *	Mammography	NR	No	Transpara	No	Retrospective study, data from Sagara Hospital Affiliated Breast Center, Japan.	NR/NR/620	2018.01–2018.10	No
Zhang et al.^72^	Mammography	NR	Yes	MVNN	No	Retrospective study, data from Digital Database for Screening Mammography.	5194/512/NR	NR	Yes
Bao et al.^73^ *	Cytology	NR	No	DL	No	Retrospective study. data from a cervical cancer screening program.	103793/NR/69906	2017.01–2018.12	No
Holmström et al.^74^ *	Cytology	NR	No	CNN	No	Retrospective study, data from a rural clinic in Kenya.	350/390/NR	2018–2019	No
Cho et al.^75^ *	Colposcopy	NR	Yes	Inception-Resnet-v2, Resnet-152	No	Retrospective study, data from three university affiliated hospitals.	675/116/NR	2015–2018	No
Bao et al.^76^ *	Cytology	NR	No	VGG16	No	Retrospective study, data from eight tertiary hospitals in China.	15083/NR/2145	2017.05–2018.10	No
Hu et al.^77^ *	Cervicography	NR	Yes	Faster R-CNN	Yes	Retrospective study, data from Guanacaste costa Rica cohort.	744/8917/NR	1993–2001	No
Hunt et al.^78^ *	Microendoscopy	NR	Yes	CNN	No	Prospectively study, data from Barretos Cancer Hospital.	870/616/NR	NR	No
Wentzensen et al.^79^ *	Cytology	NR	No	CNN4, Inception-v3	No	Retrospective study, data from Kaiser Permanente Northern California and the University of Oklahoma.	193/409/NR	2009–2014	No
Xue et al.^39^ *	Colposcopy	Yes	No	U-Net, YOLO	Yes	Retrospective study, data from six multicenter hospitals across China.	77788/23479/NR	2018.01–2018.12	No
Yu et al.^80^ *	Colposcopy	NR	No	C-GCNN, GRU	No	Retrospective study, data from First Affiliated Hospital of the University of Science and Technology of China.	3802/951/NR	2013.07–2016.09	No
Yuan et al.^40^ *	Colposcopy	Yes	No	ResNet, U-Net, MASK R-CNN	Yes	Retrospective study, data from Women’s Hospital, School of Medicine, Zhejiang University.	40194/4466/NR	2013.08–2019.05	No

*NR* not reported, *CNN* convolutional neural network, *DL* deep learning, *YOLO* you only look once, *DNN* deep neural network, *DCNN* deep convolutional neural network, *MRI* magnetic resonance imaging, *DenseNet* dense convolutional network, *MA-CNN* multiattention convolutional neural network, *VGG* visual geometry group network, *ResNet* deep residual network, *FPN* feature pyramid networks, *MVNN* multiview feature fusion neural network, *GRU* gate recurrent Unit.

*20 studies included in the meta-analysis.

Thirty-three studies utilized retrospective data. Only two studies used prospective data. Two studies also used data from open access sources. No studies reported a prespecified sample size calculation. Eight studies excluded low quality images, while 27 studies did not report anything around image quality. 11 studies performed external validation using out-of-sample dataset, while the others performed internal validation using in-sample-dataset. 12 studies compared DL algorithms against human clinicians using the same dataset. Additionally, medical imaging modalities were categorized into cytology (*n* = 4), colposcopy (*n* = 4), cervicography (*n* = 1), microendoscopy (*n* = 1), mammography (*n* = 12), ultrasound (*n* = 11), and MRI (*n* = 2).

### Pooled performance of DL algorithms

**Fig. 2:**
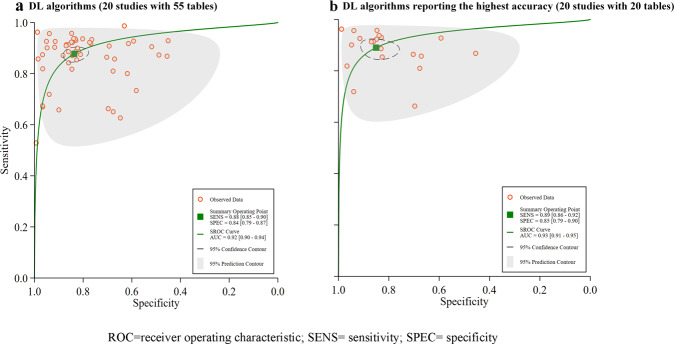
Pooled overall performance of DL algorithms. **a** Receiver operator characteristic (ROC) curves of all studies included in the meta-analysis (20 studies with 55 tables), and **b** ROC curves of studies reporting the highest accuracy (20 studies with 20 tables).

Among the 35 studies in this sample, 20 provided sufficient data to create contingency tables for calculating diagnostic performance and were therefore included for synthesis at the meta-analysis stage. Hierarchical SROC curves for these studies (i.e. 55 contingency tables) are provided in Fig. 2a. When averaging across studies, the pooled sensitivity and specificity were 88% (95% CI 85–90), and 84% (95% CI 79–87), respectively, with an AUC of 0.92 (95% CI 0.90–0.94) for all DL algorithms.

Most studies used more than one DL algorithm to report diagnostic performance, therefore we reported the highest accuracy of different DL algorithms for included studies in 20 contingency tables. The pooled sensitivity and specificity were 89% (86–92%), and 85% (79–90%), respectively, with an AUC of 0.93 (0.91–0.95). Please see Fig. 2b for further details.

### Subgroup meta-analyses

**Fig. 3:**
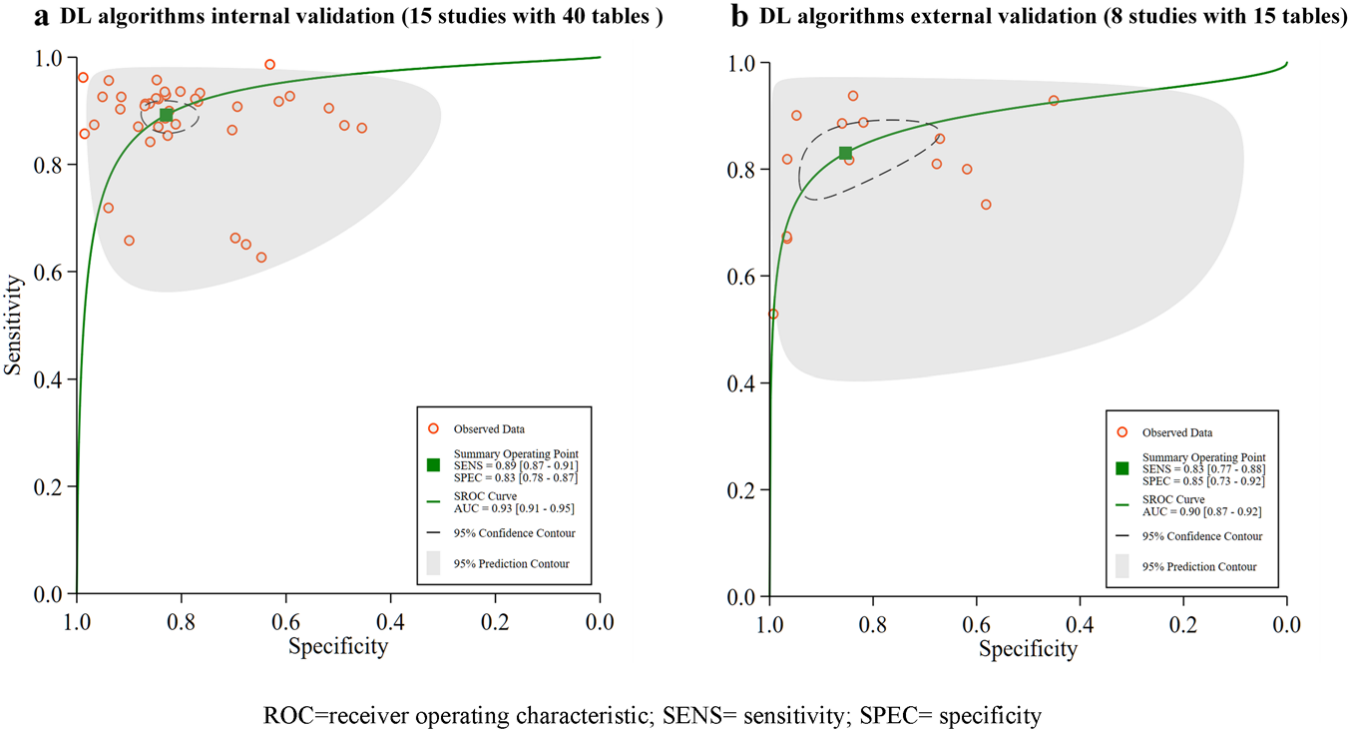
Pooled performance of DL algorithms using different validation types. **a** Receiver operator characteristic (ROC) curves of studies with internal validations (15 studies with 40 tables), **b** ROC curves of studies with external validations (8 studies with 15 tables).
II. Cancer types—10 studies with 36 contingency tables targeting breast cancer, had a pooled sensitivity of 90% (87–92%) and specificity of 85% (80–89%), with an AUC of 0.94 (0.91–0.96), see Fig. 4a. 10 studies with 19 contingency tables considered cervical cancer with a pooled sensitivity and specificity of 83% (78–88%), and 80 (70–88%), respectively, with an AUC of 0.89 (0.86–0.91), see Fig. 4b for details.

Four separate meta-analyses were conducted:

I. Validation types—15 studies with 40 contingency tables included in the meta-analysis were validated with an in-sample dataset and had a pooled sensitivity of 89% (87–91%), and pooled specificity of 83% (78–86%), with an AUC of 0.93 (0.91–0.95), see Fig. 3a for details. Only 8 studies with 15 contingency tables performed an external validation, for which the pooled sensitivity and specificity were 83% (77–88%), and 85% (73–92%), respectively, with an AUC of 0.90 (0.87–0.92), see Fig. 3b.

**Fig. 4:**
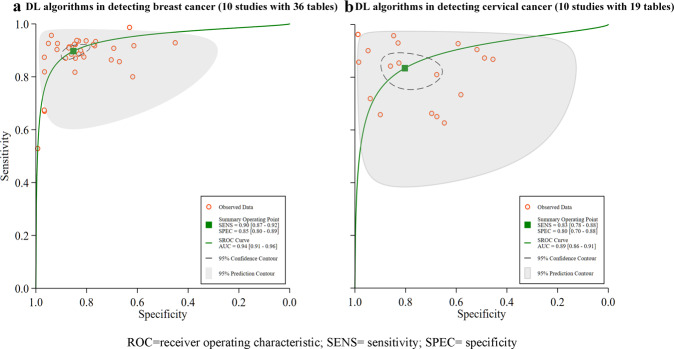
Pooled performance of DL algorithms using different cancer types. **a** Receiver operator characteristic (ROC) curves of studies in detecting breast cancer (10 studies with 36 tables), and **b** ROC curves of studies in detecting cervical cancer (10 studies with 19 tables).
III. Imaging modalities—4 mammography studies with 15 contingency tables had a pooled sensitivity of 87% (82–91%), a pooled specificity of 88% (79–93%), and with an AUC of 0.93 (0.91–0.95), see Fig. 5a. There were four ultrasound studies with 17 contingency tables with a pooled sensitivity of 91% (89–93%), pooled specificity of 85% (80–89%), and an AUC of 0.95 (0.93–0.96), see Fig. 5b. There were four cytology studies with six contingency tables which had a pooled sensitivity of 87% (82–90%), pooled specificity of 86% (68–95%), and an AUC of 0.91(0.88–0.93), Fig. 5c. There were four colposcopy studies with 11 contingency tables which had a pooled sensitivity of 78% (69–84%), pooled specificity of 78% (63–87%), and an AUC of 0.84 (0.81–0.87), see Fig. 5d.

**Fig. 5:**
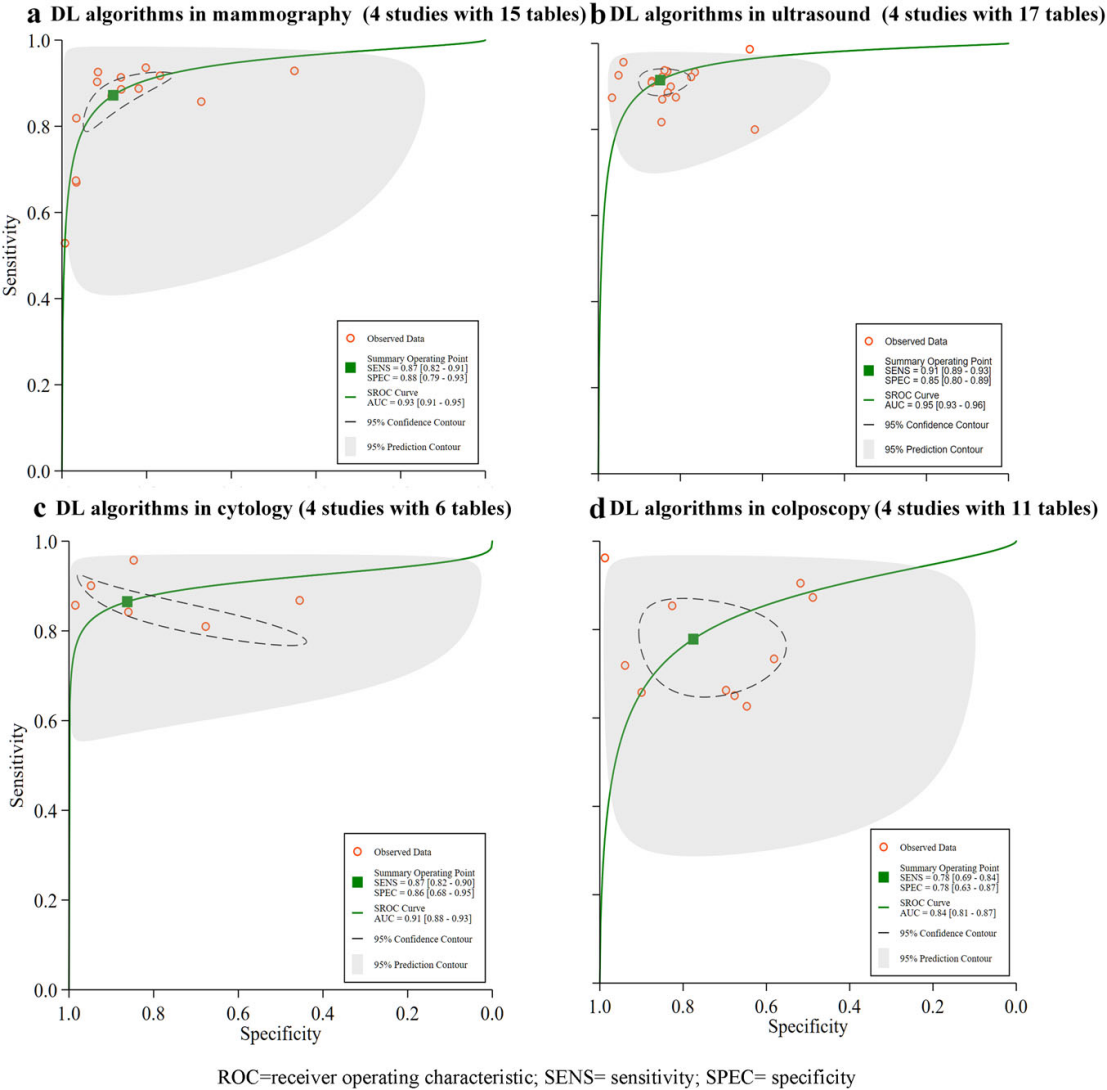
Pooled performance of DL algorithms using different imaging modalities. **a** Receiver operator characteristic (ROC) curves of studies using mammography (4 studies with 15 tables), **b** ROC curves of studies using ultrasound (4 studies with 17 tables), **c** ROC curves of studies using cytology (4 studies with 6 tables), and **d** presented ROC curves of studies using colposcopy (4 studies with 11 tables).
IV. DL algorithms versus human clinicians—of the 20 included studies, 11 studies compared diagnostic performance between DL algorithms and human clinicians using the same dataset, with 29 contingency tables for DL algorithms, and 18 contingency tables for human clinicians. The pooled sensitivity was 87% (84–90%) for DL algorithms, which human clinicians had 88% (81–93%). The pooled specificity was 83% (76–88%) for DL algorithms, and 82% (72–88%) for human clinicians. The AUC was 0.92 (0.89–0.94) for DL algorithms, and 0.92 (0.89–0.94) for human clinicians (Fig. 6a, b).

**Fig. 6:**
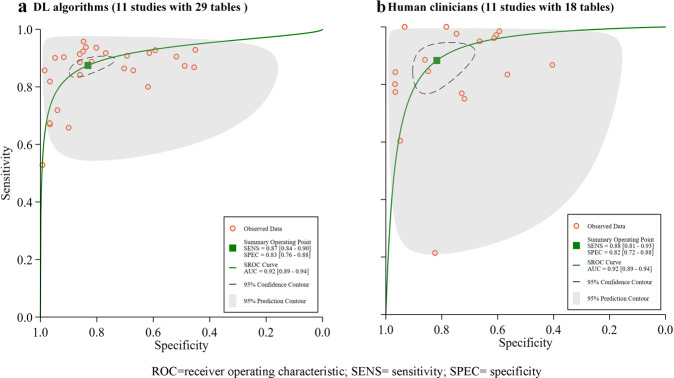
Pooled performance of DL algorithms versus human clinicians and human clinicians using the same sample. **a** Receiver operator characteristic (ROC) curves of studies using DL algorithms (11 studies with 29 tables), and **b** ROC curves of studies using human clinicians (11 studies with 18 tables).

### Heterogeneity analysis

**Fig. 7:**
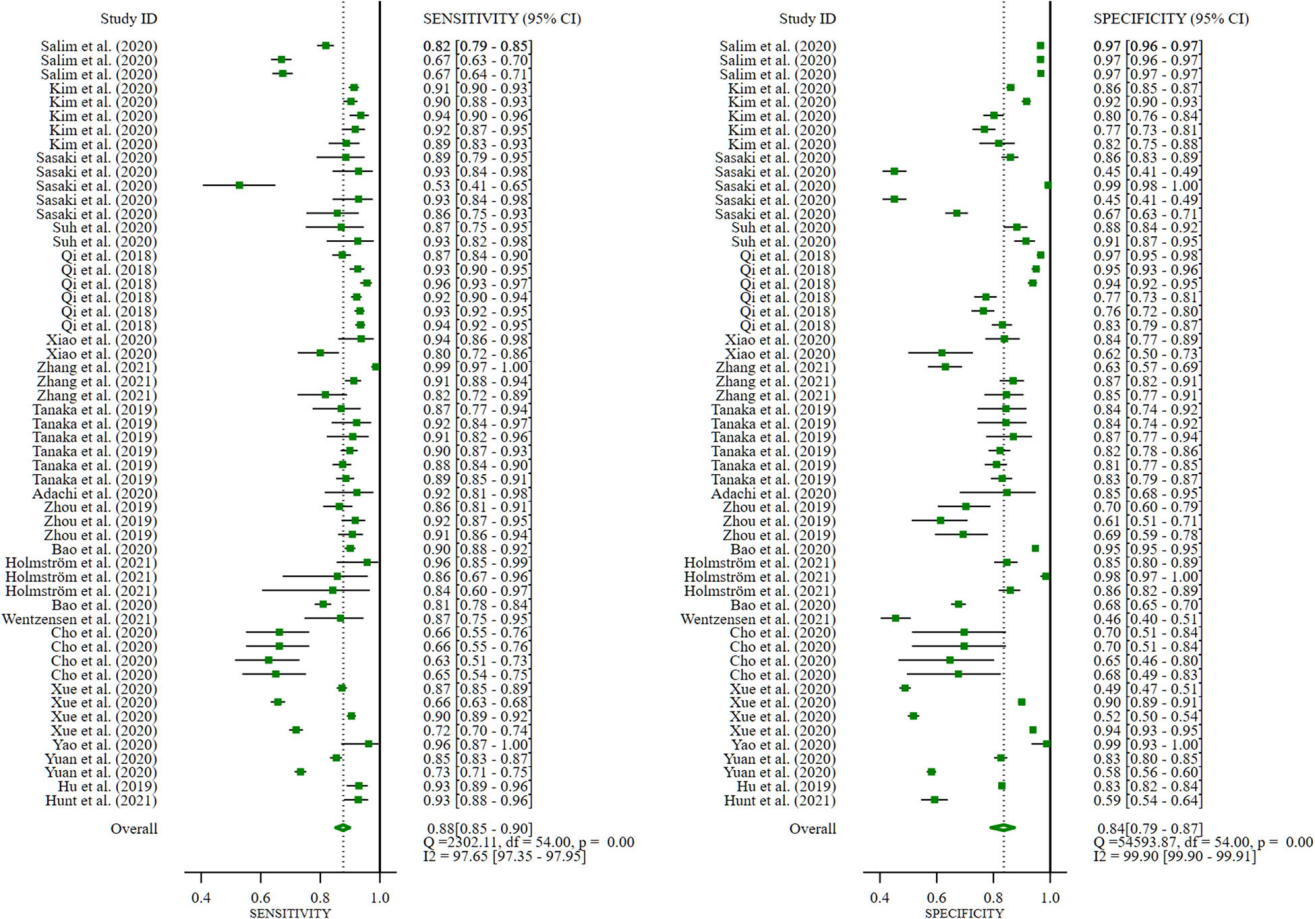
Summary estimate of pooled performance using forest plot. Data presented forest plot of studies included in the meta-analysis (20 studies).

All included studies found that DL algorithms are useful for the detection of breast and cervical cancer using medical imaging when compared with histopathological analysis, as the gold standard; however, extreme heterogeneity was observed. Sensitivity (SE) had an *I*^*2*^ = 97.65%, while specificity (SP) had *I*^*2*^ = 99.90 (*p* < 0.0001), see Fig. 7.

A funnel plot was produced to assess publication bias. The *p* value of 0.41 suggests there is no publication bias although studies were widely dispersed around the regression line. See Supplementary Fig. 3 for further details. In order to identify the source/sources of such extreme heterogeneity we conducted subgroup analysis, and found:

I. Validation types—Internal validation (SE, *I*^*2*^ = 97.60%, SP, *I*^*2*^ = 99.19, *p* < 0.0001), and external validation (SE, *I*^*2*^ = 96.15%, SP, *I*^*2*^ = 99.96, *p* < 0.0001). See Supplementary Fig. 4.
II. Cancer types of DL algorithms included breast cancer (SE, *I*^*2*^ = 95.84%, SP, *I*^*2*^ = 99.86 *p* < 0.0001) and cervical cancer (SE, *I*^*2*^ = 98.16%, SP, *I*^*2*^ = 99.89, *p* < 0.0001). Please see Supplementary Fig. 5 for further details.
III. Imaging modalities including mammography (SE, *I*^*2*^ = 97.01%, SP, *I*^*2*^ = 99.93, *p* < 0.0001), and ultrasound (SE, *I*^*2*^ = 86.49%, SP, *I*^*2*^ = 96.06, *p* < 0.0001), cytology (SE, *I*^*2*^ = 89.97%, SP, *I*^*2*^ = 99.90, *p* < 0.0001), and colposcopy (SE, *I*^*2*^ = 98.12%, SP, *I*^*2*^ = 99.59, *p* < 0.0001), see Supplementary Fig. 6.

However, heterogeneity was not aligned to a specific subgroup, nor was it reduced to an acceptable level, with all subgroup *I*^*2*^ values remained high. Therefore, we could infer whether different validation types, cancer types, and imaging modalities were likely to have influenced DL algorithm performances for detecting breast and cervical cancer.

To further investigate this finding, we performed meta-regression analysis with these covariates (see Supplementary Table 1). The results highlighted a statistically significant difference, which is line with sub-group and meta-analytical sensitivity analyses.

### Quality assessment

The quality of the included studies was assessed using QUADAS-2 and a summary of findings has been provided with an appropriate diagram in the supplementary materials as Supplementary Fig. 1. A detailed assessment for each item based on the domain of risk of bias and concern of applicability has also been provided as Supplementary Fig. 2. For the patient selection domain of risk of bias, 13 studies were considered high or unclear risk of bias due to unreported inclusion criteria or exclusion criteria, and improper exclusion. For the index test domain, only one studies was considered high or at unclear risk of bias due to having no predefined threshold, whereas the others were considered at low risk of bias.

For the reference standard domain, three studies were considered at high or unclear risk of bias due to reference standard inconsistencies. There was no mention of whether the threshold was determined in advance and whether blinding was implemented. For the flow and timing domain, five studies were considered high or with an unclear risk of bias because the authors had not mentioned whether there was an appropriate time gap or whether it was based on the same gold standard.

In the applicability concern domain, 12 studies were considered to have high or unclear applicability in patient selection. One study also had unclear applicability in the reference standard domain, with no applicability concern in the index test domain.

## Discussion

Artificial Intelligence in medical imaging is without question improving however, we must subject emerging knowledge to the same rigorous testing we would for any other diagnostic procedure. Deep learning could reduce the over-reliance of experienced clinicians and could, with relative ease, be extended to rural communities and LMICs. While this relatively inexpensive approach may help to bridge inequality gaps across healthcare systems generally, evidence is increasingly highlighting the value of deep learning in cancer diagnostics and care. Within the field of female cancer diagnosis, one of the representative technologies is computer-assisted cytology image diagnosis such as the FDA-approved PAPNET and AutoPap systems, which dates back to at least the 1970s^22^. While rapid progress in AI technology is made, they are also becoming an increasingly important element involved in automated image-based cytology analysis systems. These technologies have the potential to reduce the amount of time spent and improve cytologics during the reading process. Here, we attempted to ascertain which is the most accurate and reliable detection technology presently available in the field of breast and cervical cancer diagnostics.

A systematic search for pertinent articles identified three systematic reviews with meta-analyses which investigated DL algorithms in medical imaging. However, these were in diverse domains which make it difficult to compare directly with the present review. For example, Liu et al. ^23^ found that DL algorithm performance in medical imaging might be equivalent to healthcare professors. However, only breast and dermatological cancers were analyzed with more than three studies, which not only inhibits generalizability but highlights the need for further DL algorithm performance research in the field of medical imaging. In identifying pathologies, Aggarwal et al. ^24^ found that DL algorithms have high diagnostic performance. However, the authors also found high heterogeneity which was attributed to combining distinct methods and perhaps through unspecified terms. They concluded that we need to be cautious when considering the diagnostic accuracy of DL algorithms and that there is a need to develop (and apply) AI guidelines. This was also apparent in this study and therefore we would reiterate this sentiment.

While the findings from the aforementioned studies are incredibly valuable, at present there is a need to expand upon the emerging knowledge-base for metastatic tumor diagnosis. The only other review in this field was conducted by Zheng et al. ^25^ who found that DL algorithms are beneficial in radiological imaging with equivalent, or in some instances better performance than healthcare professionals. Although again, there were methodological deficiencies which must be considered before we adopt these technologies into clinical practice. Also, we must strive to identify the best available DL algorithm and then develop it to enhance identification and reduce the number of false positives and false negatives beyond that which is humanly possible. As such, we need to continue to use systematic reviews to identify gaps in research and we should not only consider technology-specific reviews, but also disease-specific systematic reviews. Of course, DL algorithms are in an almost constant state of development but the purpose of this study was to critically appraise potential issues with study methods and reporting standards. By doing so, we hoped to make recommendations and to drive further research in this field so that the most effective technology is adopted into clinical practice sooner rather than later.

This systematic review with meta-analysis suggests that deep learning algorithms can be used for the detection of breast and cervical cancer using medical imaging. Evidence also suggests that while the deep learning algorithms are not yet superior, nor are they inferior in terms of performance when compared to clinicians. Acceptable diagnostic performance with analogous deep learning algorithms was observed in both breast and cervical cancer despite having dissimilar workflows with different imaging modalities. This finding also suggests that these algorithms could be deployed across both breast or cervical imaging, and potentially across all types of cancer which utilize imaging technologies to identify cases early. However, we must also critically consider some of the issues which emerged during our systematic analysis of this evidence base.

Overall, there were very few prospective studies and few clinical trials. In fact, most included studies were retrospective studies which may be the case because of the relative newness of DL algorithms in medical imaging. However, the data sources used were from either pre-existing electronic medical records or online open-access databases, which were not explicitly intended for algorithmic analysis in real clinical settings. Of course, we must first test these technologies using retrospective datasets to see whether they are appropriate and with a view to modifying and enhancing accuracy perhaps for specific populations or for specific types of cancer. We also encourage more prospective DL studies in the future. If possible, we should investigate the potential rules of breast or cervical images through more prospective studies, and identify possible image feature correlations and diagnostic logic for risk predictions. Most studies constructed and trained algorithms using small labeled breast or cervical images, with labels which were rarely quality-checked by a clinical specialist. This design fault is likely to have created ambiguous ground-truth inputs which may have caused unintended adverse model effects. Of course, the knock-on effect is that there is likely to be diagnostic inaccuracies through unidentified biases. This is certainly an issue which should be considered when designing future deep learning-based studies.

It is important to note that no matter how well-constructed an algorithm is, its diagnostic performance depends largely upon the volume of raw data and quality^26^. Most studies included in this systematic review mentioned a data augmentation method which adopted some form of affine image transformations strategy e.g. translational, rotation or flipping, in order to compensate for data deficiencies. This, one could argue, is symptomatic of the paucity of annotated datasets for model training, and prospective studies for model validation. Though fortunately, there has been a substantial increase in the number of openly available datasets around cervical or breast cancer. However, given the necessity for this research, one would like to see institutions collaborating more frequently to establish cloud sharing platforms which would increase the availability (and breadth) of annotated datasets. Moreover, training DL algorithms requires reliable, high-quality image inputs, which may not be readily available, as some pre-analytical factors such as incorrect specimen preparation and processing, unstandardized image digitalization acquisition, improper device calibration and maintenance could lower image quality. Complete standardization of all procedures and reagents in clinical practice is required to optimally prepare pre-analytical image inputs in order to develop more robust and accurate DL algorithms. Having these would drive developments in this field and would benefit clinical practice, perhaps serving as a cost-effective replacement diagnostic tool or an initial method of risk categorization. Although, this is beyond the scope of this study and would require further research to consider this in detail.

Of the 35 included studies, only 11 studies performed external validation, which means that an assessment of DL model performance was conducted with either an out-of-sample dataset or with an open-access dataset. Indeed, most of the studies included here split a single sample by either randomly and non-randomly assigning individuals’ data from one center into one development dataset or the other internal validations dataset. We found that studies with internal validation were higher than externally validated studies for early detection of cervical and breast cancer. However, this was to be expected because using an internal dataset to validate models is more likely homogenous and may lead to an overestimated diagnostic performance. This finding highlights the need for out-of-sample external validation in all predictive models. A possible method for improving external validation would be to establish an alliance of institutions wherein trained deep learning algorithms are shared and performances tested, externally. This might provide insight into subgroups and variations between various ethnic groups although we would also need to maintain patient anonymity and security, as several researchers have previously noted^27,28^.

Most of the studies that were retrospective using narrowly defined binary or multi-class tests focusing on the diagnostic performance in the field of DL algorithms rather than clinical practice. This is a direct consequence of poor reporting, and the lack of real-world prospective clinical practice, which has resulted in inadequate data availability and therefore may limit our ability to gauge the applicability of these DL algorithms to clinical settings. Accordingly, there is uncertainty around the estimates of diagnostic performance provided in our meta-analysis and adherence levels should be interpreted with caution.

Recently, several AI-related method guides have been published, with many still under development^29,30^. We found most of the included studies we analyzed were probably conceived or performed before these guidelines were available. Therefore, it is reasonable to assume that design features, reporting adequacy and transparency of studies used to evaluate the diagnostic performance of DL algorithms will be improved in the future. Even though, our findings suggest that DL is not inferior in terms of performance compared to clinicians for the early detection of breast or cervical cancer, this is based on relatively few studies. Therefore, the uncertainty which exists is, at least in part, due to the in silico context in which clinicians are being evaluated.

We should also acknowledge that most of the current DL studies are publications of positive results. We must be aware that this may be a form of researcher-based reporting bias (rather than publication-based bias), which is likely to skew the dataset and adds complexity to comparison between DL algorithms and clinicians^31,32^. Differences in reference standard definitions, grader capabilities (i.e. the degrees of expertise), imaging modalities and detection thresholds for classification of early breast or cervical cancer also make direct comparisons between studies and algorithms very difficult. Furthermore, non-trivial applications of DL models in the healthcare setting will need clinicians to optimize clinical workflow integration. However, we found only two of studies which mentioned DL versus clinicians and versus DL combined with clinicians. This hindered our meta-analysis of DL algorithms but highlighted the need for strict and reliable assessment of DL performance in real clinical settings. Indeed, the scientific discourse should change from DL versus clinicians dichotomy to a more realistic DL-clinician combination, which would improve workflows.

35 studies met the eligibility criteria for the systematic review, yet only 20 studies could be used to develop contingency tables. Some DL algorithm studies from computer science journals only reported precision, dice coefficient, F1 score, recall, and competition performance metric. Whereas indicators such as AUC, accuracy, sensitivity, and specificity are more familiar to healthcare professionals^25^. Bridging the gap between computer sciences research would seem prudent if we are to manage interdepartmental research and the transition to a more digitized healthcare system. Moreover, we found the term “validation” is used causally in DL model studies. Some authors used it for assessing the diagnostic performance of the final algorithm, others defined it as a dataset for model tuning during the development process. This confuses readers and makes it difficult to judge the function of datasets. We combined experts’ opinions^33^, and proposed to distinguish datasets used in the development and validation of DL algorithms. In keeping with the language used for nomogram development, a dataset for training the model should be named ‘training set’, while datasets used for tuning should be referred to as the ‘tuning set’. Likewise, during the validation phase, the hold-back subset split from the entire dataset should be referred to a ‘internal’ validation, which is the same condition/image types as the training set. While a completely independent dataset for our-of-sample validation should be referred to as ‘external’ validation^34^.

Most of the issues discussed here could be avoided through more robust designs and high-quality reporting, although several hurdles must be overcome before DL algorithms are used in practice for breast and cervical cancer identification. The black box nature of DL models without clear interpretability of the basis for the clinical situations is a well-recognized challenge. For example, a clinician considering whether breast nodules represent breast cancer based on mammographic images for a series of judgement criteria. Therefore, a clinician developing a clear rationale for a proposed diagnosis maybe the desired state. Whereas, having a DL model which merely states the diagnosis may be viewed with more skepticism. Scientists have actively investigated possible methods for inspecting and explaining algorithmic decisions. An important example is the use of salience or heat maps to provide the location of salient lesion features within the image rather than defining the lesion characteristics themselves^35,36^. This raises questions around human-technology interactions, and particularly around transparency and patient-practitioner communications which ought to be studied in conjunction with DL modeling in medical imaging.

Another common problem limiting DL algorithms is model generalizability. There may be potential factors in the training data that would affect the performance of DL models in different data distribution settings^28^. For example, a model only trained in US may not perform well in Asia because a model trained using data from predominantly caucasian patients may not perform well across other ethnicities. One solution to improve generalizability and reduce bias is to conduct large, multicenter studies which can enable the analysis of nationalities, ethnicities, hospital specifics, and population distribution characteristics^37^. Societal biases can also affect the performance of DL models and of course, bias exists in DL algorithms because a training dataset may not include appropriate proportions of minority groups. For example, a DL algorithm for melanoma diagnosis in dermatological study may lack diversity in terms of skin color and genomic data, but this may also cause an under-representation of minority groups^38^. To eliminate embedded prejudice, efforts should be made to carry out DL algorithm research which provides a more realistic representation of global populations.

As we have seen, the included studies were mostly retrospective with extensive variation in methods and reporting. More high-quality studies such as prospective studies and clinical trials are needed to enhance the current evidence base. We also focused on DL algorithms for breast and cervical cancer detection using medical imaging. Therefore, we made no attempt to generalize our findings to other types of AI, such as conventional machine learning models. While there were a reasonable number of studies for this meta-analysis, the number of studies of each imaging modality was limited like cytology or colposcopy, Therefore, the results of the subgroup analyses around imaging modality needs to be interpreted with caution. We also selected only studies in which histopathology was used as the reference standard. Consequently, some DL studies that may have shown promise but did not have confirmatory histopathologic results, were excluded. Even though the publication bias was not identified through funnel plot analysis in Supplementary Fig. 3 based on data extracted from 20 studies, the lack of prospective studies and the potential absence of studies with negative results can cause bias. As such, we would encourage deep learning researchers in medical imaging to report studies which do not reject the null hypothesis because this will ensure evidence clusters around true effect estimates.

It remains necessary to promote deep learning in medical imaging studies for breast or cervical cancer detection. However, we suggest improving breast and cervical data quality and establishing unified standards. Developing DL algorithms needs to feed on reliable and high-quality images tagged with appropriate histopathological labels. Likewise, it is important to establish unified standards to improve the quality of the digital image-production, the collection process, imaging reports, and final histopathological diagnosis. Combining DL algorithm results with other biomarkers may prove useful to improve risk discrimination for breast or cervical cancer detection. An example would be a DL model for cervical imaging that combines with additional clinical information i.e. cytology and HPV typing, which could improve overall diagnostic performance^39,40^. Secondly, we need to improve the error correction ability and DL algorithm compatibility. Prophase developing DL algorithms are more generalizable and less susceptible to bias but may require larger and multicenter datasets which incorporate diverse nationalities and ethnicities, as well as those with different socioeconomic status etc., if we are to implement algorithms into real-world settings.

This also highlights the need for international reporting guidelines for DL algorithms in medical imaging. Existing reporting guidelines such as STARD^41^ for diagnostic accuracy studies, and TRIPOD^42^ for conventional prediction models are not available to DL model study. The recent publication of CONSORT-AI^43^ and SPIRIT-AI^44^ guidelines are welcomed but we await disease-specific DL guidelines. Furthermore, we would encourage organizations to develop diverse teams, combining computer scientists and clinicians to solve clinical problems using DL algorithms. Even though DL algorithms appear like black boxes with unexplainable decision-making outputs, these technologies need to be discussed for development and require additional clinical information^45,46^. Finally, medical computer vision algorithms do not exist in a vacuum, we must integrate DL algorithms into routine clinical workflows and across entire healthcare systems to assist doctors and augment decision-making. Therefore, it is crucial that clinicians understand the information each algorithm provides and how this can be integrated into clinical decisions which enhance efficiency without absorbing resources. For any algorithm to be incorporated into existing workflows it has to be robust, and scientifically validated for clinical and personal utility.

We tentatively suggest that DL algorithms could be useful for detecting breast and cervical cancer using medical imaging, with equivalent performance to human clinicians, in terms of sensitivity and specificity. However, this finding is based on poor study designs and reporting which could lead to bias and overestimating algorithmic performances. Standardized guidelines around study methods and reporting are needed to improve the quality of DL model research. This may help to facilitate the transition into clinical practice although further research is required.

## Methods

### Protocol registration and study design

The study protocol was registered with the PROSPERO International register of systematic reviews, number CRD42021252379. The study was conducted according to the preferred reporting items for systematic reviews and meta-analyses (PRISMA) guidelines^47^. No ethical approval or informed consent was required for the current systematic review and meta-analysis.

### Search strategy and eligibility criteria

In this study, we searched Medline, Embase, IEEE and the Cochrane library until April 2021. No restrictions were applied around regions, languages, or publication types; however, letters, scientific reports, conference abstracts, and narrative reviews were excluded. The full search strategy for each database was developed in collaboration with a group of experienced clinicians and medical researchers. Please see Supplementary Note 1 for further details.

Eligibility assessment was conducted by two independent investigators, who screened titles and abstracts, and selected all relevant citations for full-text review. Disagreements were resolved through discussion with another collaborator. We included studies that reported the diagnostic performance of a DL model/s for the early detection of breast or cervical cancer using medical imaging. Studies reporting any diagnostic outcome, such as accuracy, sensitivity, and specificity etc., could be included. There was no restriction on participant characteristics, type of imaging modality or the intended context for using DL models.

Only histopathology was accepted as the study reference standard. As such, imperfect ground truths, such as expert opinion or consensus, and other clinical testing were rejected. Likewise, medical waveform data or investigations into the performance of image segmentation were excluded because these could not be synthesized with histopathological data. Animals’ studies or non-human samples were also excluded and duplicates were removed. The primary outcomes were various diagnostic performance metrics. Secondary analysis included and assessment of study methodologies and reporting standards.

### Data extraction

Two investigators independently extracted study characteristics and diagnostic performance data using predetermined data extraction sheet. Again, uncertainties were resolved by a third investigator. Binary diagnostic accuracy data were extracted directly into contingency tables which included true-positives, false-positives, true-negatives, and false-negatives. These were then used to calculate pooled sensitivity, pooled specificity, and other metrics. If a study provided multiple contingency tables for the same or for different DL algorithms, we assumed that they were independent of each other.

### Quality assessment

The risk of bias and applicability concerns of the included studies were assessed by the three investigators using the quality assessment of diagnostic accuracy studies 2 (QUADAS-2) tool^48^.

### Statistical analysis

Hierarchical summary receiver operating characteristic (SROC) curves were used to assess the diagnostic performance of DL algorithms. 95% confidence intervals (CI) and prediction regions were generated around averaged sensitivity, specificity, and AUCs estimates in SROC figures. Further meta-analysis was performed to report the best accuracy in studies with multiple DL algorithms from contingency tables. Heterogeneity was assessed using the *I*^2^ statistic. We also conducted the subgroup meta-analyses and regression analyses to explore potential sources of heterogeneity. The random effects model was implemented because of the assumed differences between studies. Publication bias was assessed visually using funnel plots.

Four separate meta-analyses were conducted: (1) according to validation type, DL algorithms were categorized as either internal or external. Internal validation meant that studies were validated using an in-sample-dataset, while external validation studies were validated using an out-of-sample dataset; (2) according to cancer type i.e., breast or cervical cancer; (3) according to imaging modalities, such as mammography, ultrasound, cytology, and colposcopy, etc; (4) according to the pooled performance for DL algorithms versus human clinicians using the same dataset.

Meta-analysis was only performed where there were more than or equal to three original studies. STATA (version 15.1), and SAS (version 9.4) were for data analyses. The threshold for statistical significance was set at *p* < 0.05, and all tests were two-sides.

### Reporting Summary

Further information on research design is available in the Nature Research Reporting Summary linked to this article.

## Supplementary information

SUPPLEMENTAL MATERIAL

Reporting Summary

## Data Availability

The search strategy and aggregated data contributing to the meta-analysis is available in the appendix.
