# Supplementary material for: Deep learning in image-based breast and cervical cancer detection: a systematic review and meta-analysis
Source: NPJ Digit Med. 2022 Feb 15;5:19. doi: 10.1038/s41746-022-00559-z (PMC8847584; doi:10.1038/s41746-022-00559-z)
Supplement: Supplementary file 1 — SUPPLEMENTAL MATERIAL [file 41746_2022_559_MOESM1_ESM.pdf]

Supplementary Online Content

Deep learning in image-based breast and cervical cancer detection: a systematic review and meta-analysis

Supplementary Figure 1: QUADAS-2 summary plot.
Risk of bias and applicability concerns summary about each QUADAS-2 domain presented as percentages across the 35 included studies.

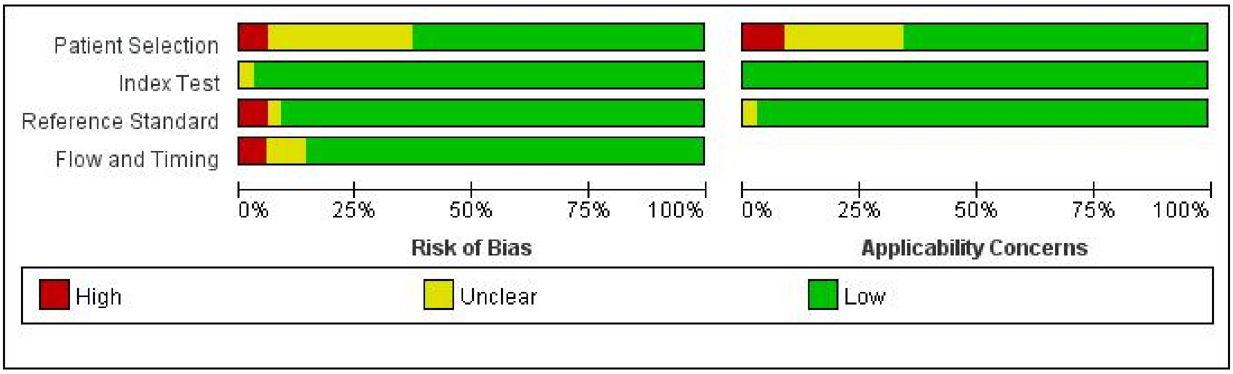

Supplementary Figure 2: Risk of bias and concern of applicability for each item in included studies.

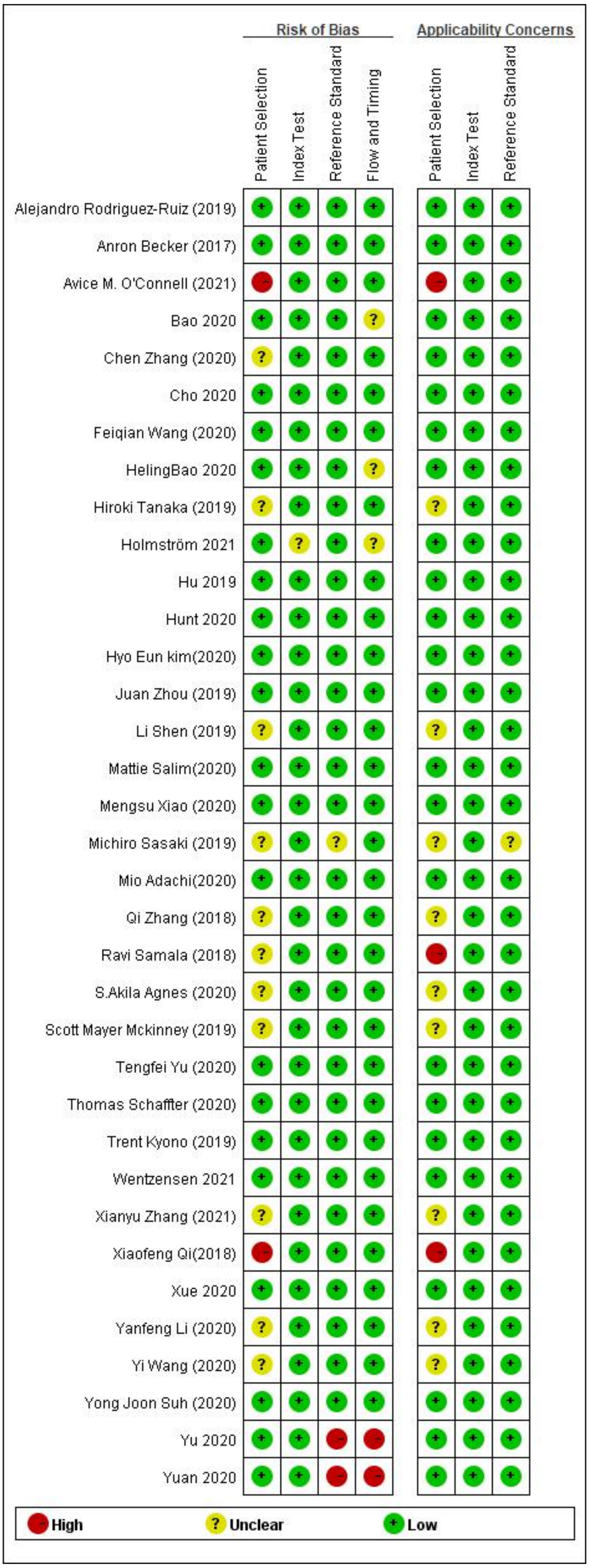

Supplementary Figure 3: Publication bias.

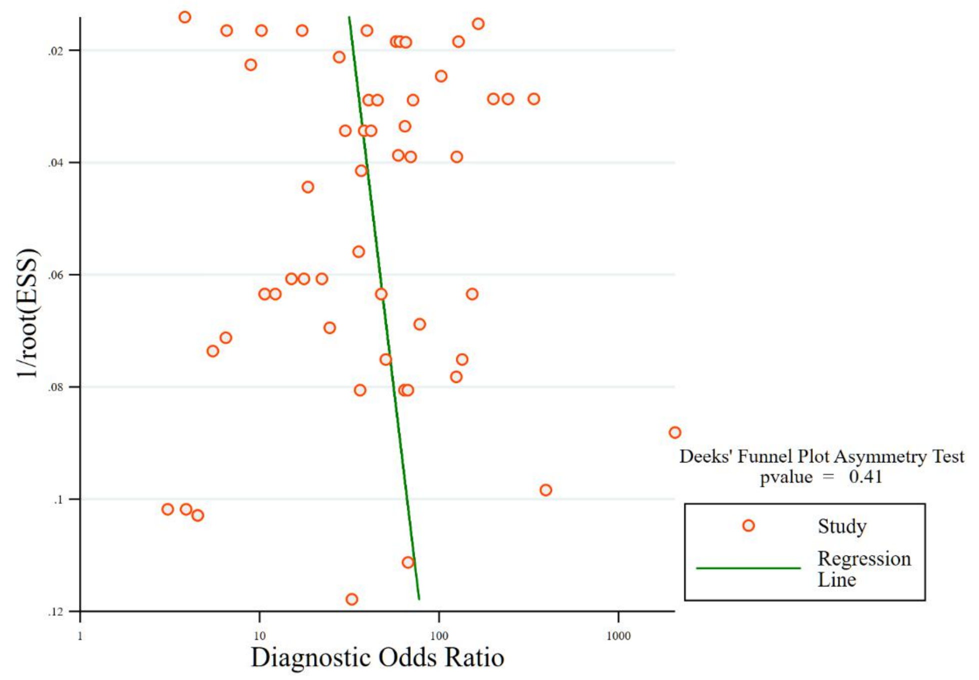

Funnel plots suggested there was no publication bias

Supplementary Table 1: Meta-regression result.

Covariates	Subgroup	LRTChi2	I^2	*P* value
Disease	Breast cancer	10.07	80%	<0.01
	Cervical cancer			
Image type	Mammogram	8.49	76%	<0.01
	Ultrasound			
	MRI			
	Cytology			
	Colposcopy			
	Cervicography			
	Microendoscopy			
External validation	Yes	26.02	92%	<0.01
	No			

Supplementary Figure 4: Forest plot of different validation types (internal or external).

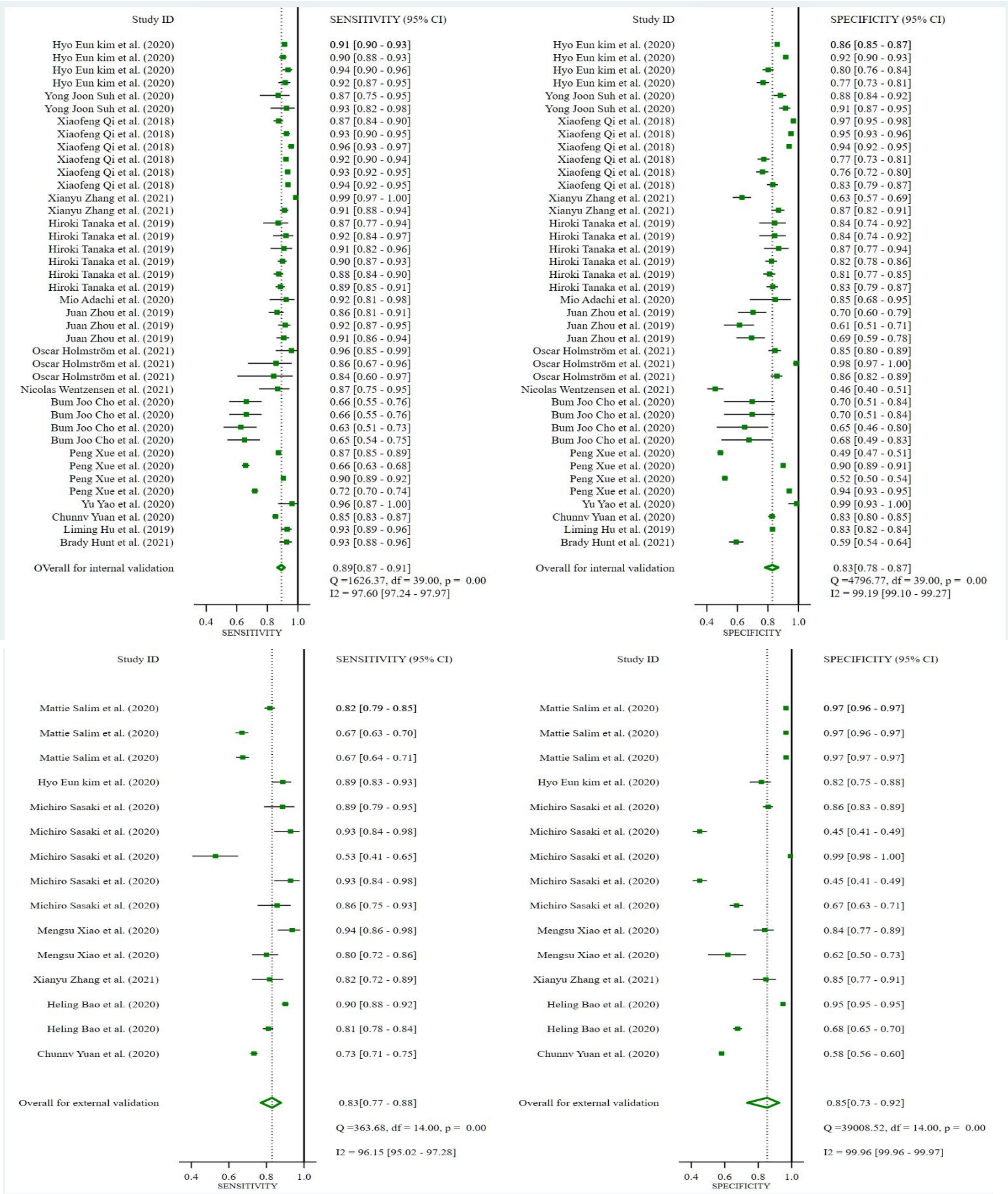

Supplementary Figure 5: Forest plot of different cancer types (breast or cervical cancer).

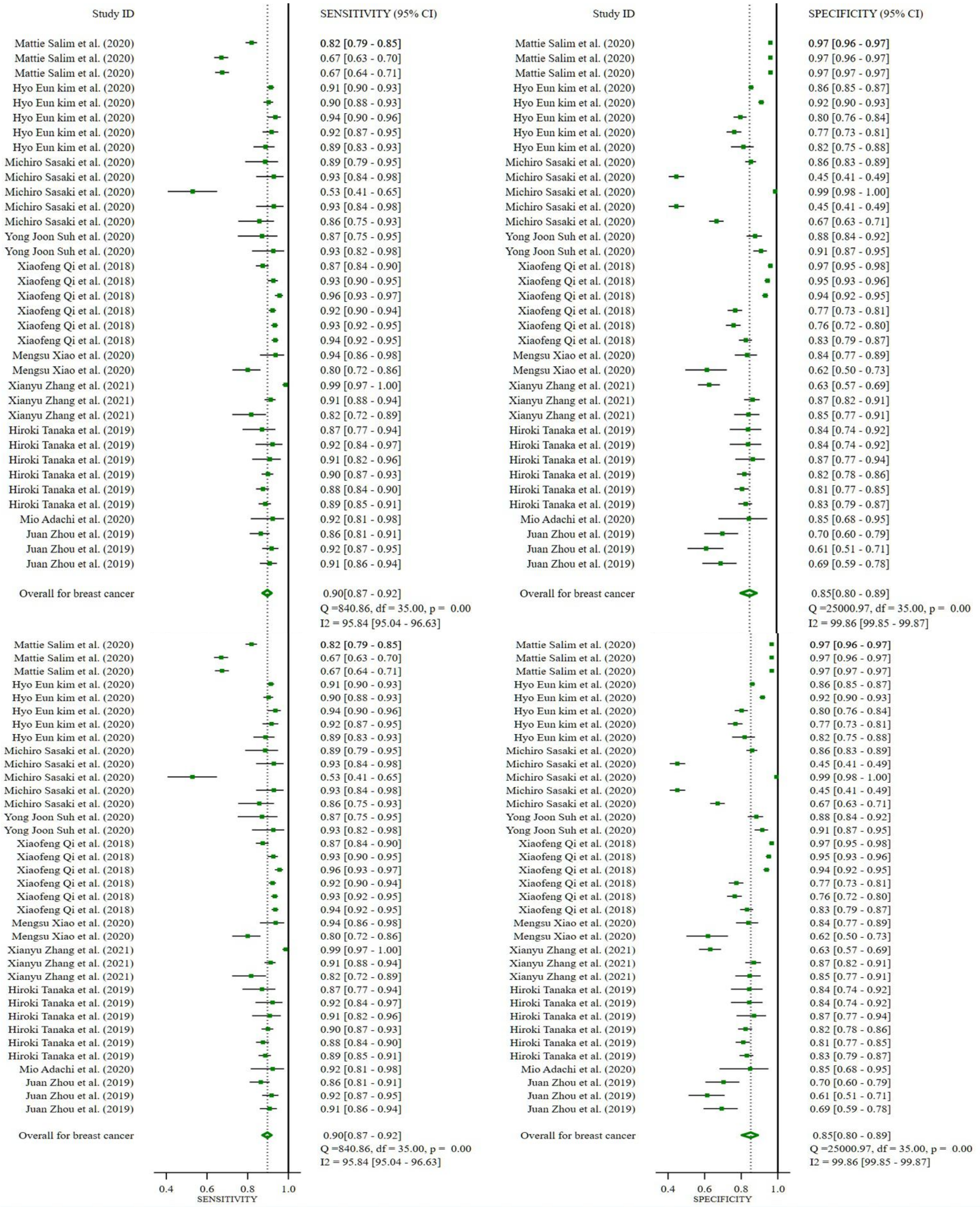

Supplementary Figure 6: Forest plot of different imaging modalities (mammography, ultrasound, cytology, and colposcopy).

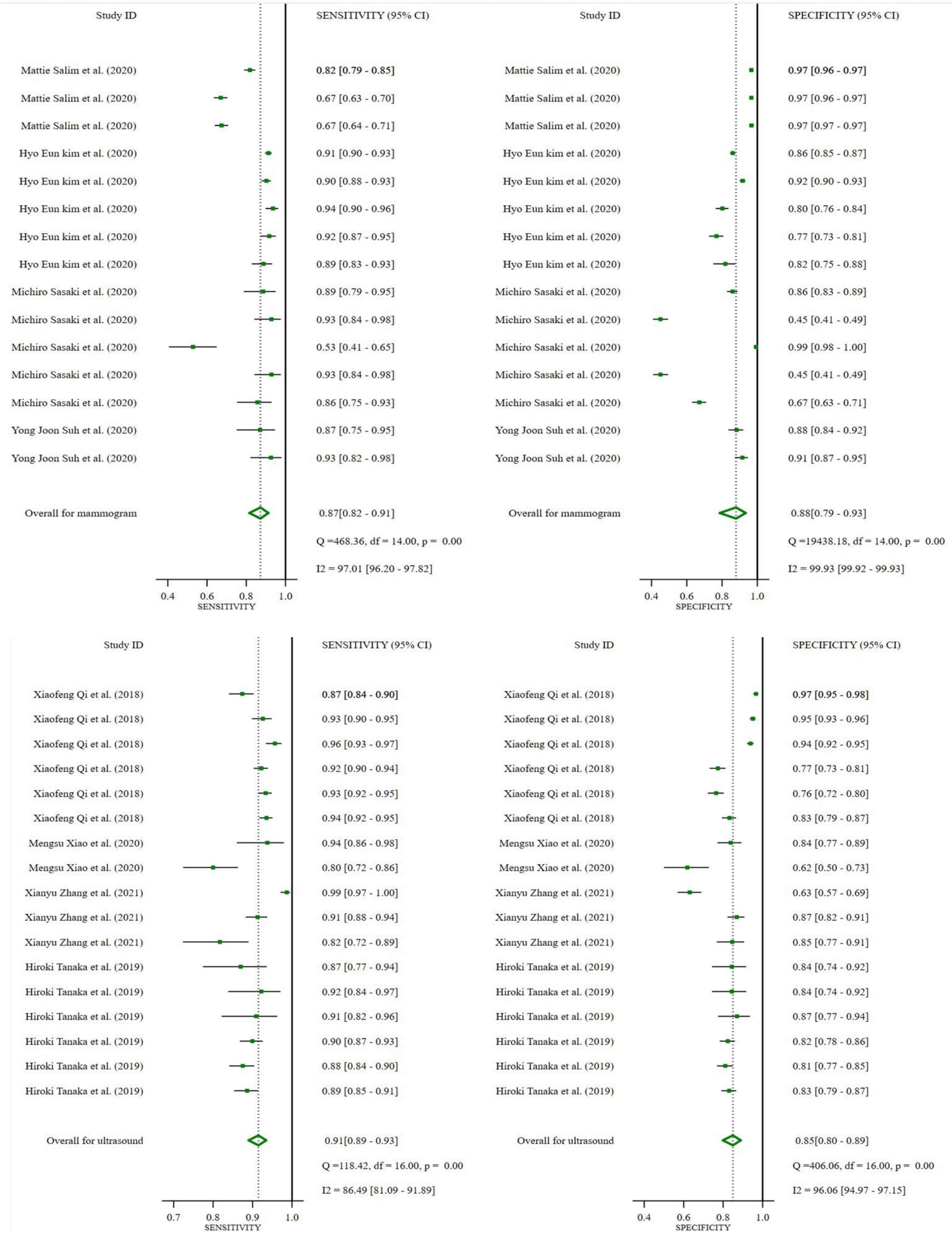

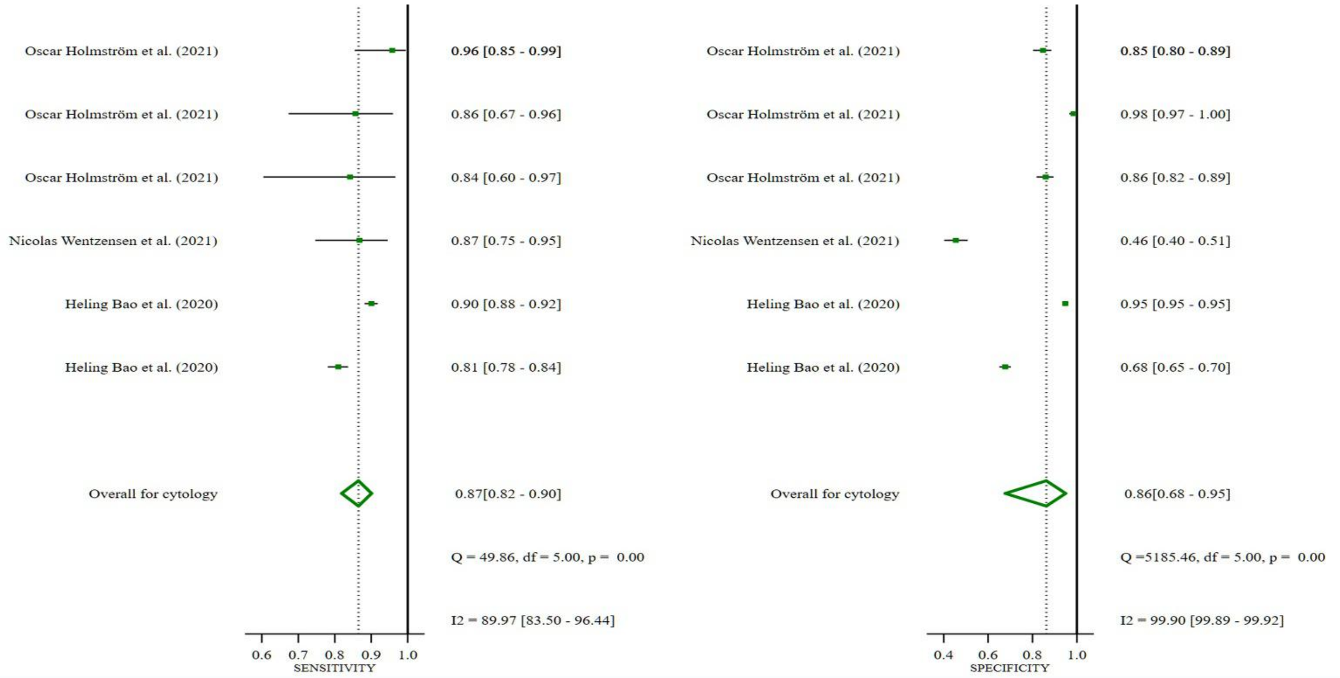
Oscar Holmström et al. (2021)
0.96 [0.85 - 0.99]
Oscar Holmström et al. (2021)
0.86 [0.67 - 0.96]
Oscar Holmström et al. (2021)
0.84 [0.60 - 0.97]
Nicolas Wentzensen et al. (2021)
0.87 [0.75 - 0.95]
Heling Bao et al. (2020)
0.90 [0.88 - 0.92]
Heling Bao et al. (2020)
0.81 [0.78 - 0.84]
Overall for cytology
0.87[0.82 - 0.90]
Q = 49.86, df = 5.00, p = 0.00
I2 = 89.97 [83.50 - 96.44]
0.6 0.7 0.8 0.9 1.0
SENSITIVITY
Oscar Holmström et al. (2021)
0.85 [0.80 - 0.89]
Oscar Holmström et al. (2021)
0.98 [0.97 - 1.00]
Oscar Holmström et al. (2021)
0.86 [0.82 - 0.89]
Nicolas Wentzensen et al. (2021)
0.46 [0.40 - 0.51]
Heling Bao et al. (2020)
0.95 [0.95 - 0.95]
Heling Bao et al. (2020)
0.68 [0.65 - 0.70]
Overall for cytology
0.86[0.68 - 0.95]
Q =5185.46, df = 5.00, p = 0.00
I2 = 99.90 [99.89 - 99.92]
0.4 0.6 0.8 1.0
SPECIFICITY

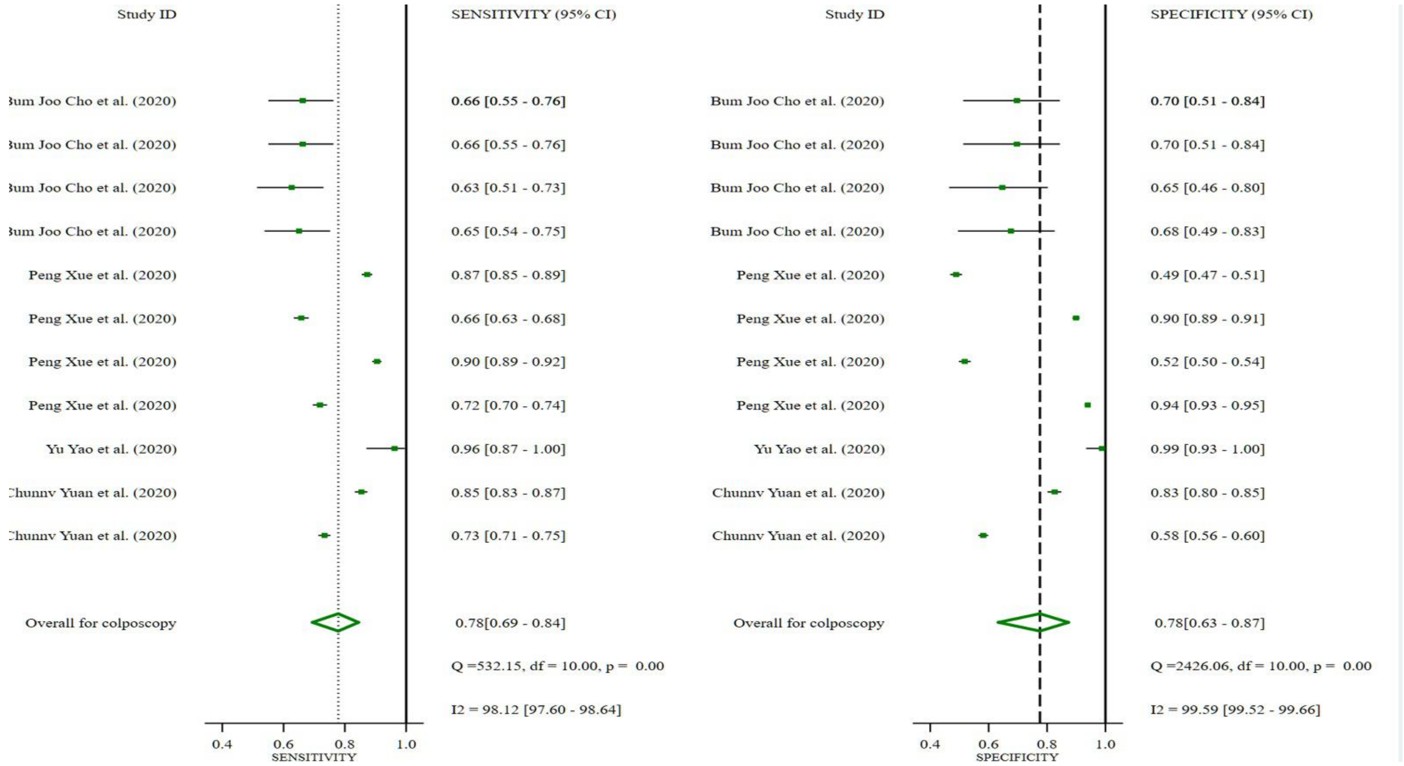
Study ID
SENSITIVITY (95% CI)
Bum Joo Cho et al. (2020)
0.66 [0.55 - 0.76]
Bum Joo Cho et al. (2020)
0.66 [0.55 - 0.76]
Bum Joo Cho et al. (2020)
0.63 [0.51 - 0.73]
Bum Joo Cho et al. (2020)
0.65 [0.54 - 0.75]
Peng Xue et al. (2020)
0.87 [0.85 - 0.89]
Peng Xue et al. (2020)
0.66 [0.63 - 0.68]
Peng Xue et al. (2020)
0.90 [0.89 - 0.92]
Peng Xue et al. (2020)
0.72 [0.70 - 0.74]
Yu Yao et al. (2020)
0.96 [0.87 - 1.00]
Chunnv Yuan et al. (2020)
0.85 [0.83 - 0.87]
Chunnv Yuan et al. (2020)
0.73 [0.71 - 0.75]
Overall for colposcopy
0.78[0.69 - 0.84]
Q =532.15, df = 10.00, p = 0.00
I2 = 98.12 [97.60 - 98.64]
0.4 0.6 0.8 1.0
SENSITIVITY
Study ID
SPECIFICITY (95% CI)
Bum Joo Cho et al. (2020)
0.70 [0.51 - 0.84]
Bum Joo Cho et al. (2020)
0.70 [0.51 - 0.84]
Bum Joo Cho et al. (2020)
0.65 [0.46 - 0.80]
Bum Joo Cho et al. (2020)
0.68 [0.49 - 0.83]
Peng Xue et al. (2020)
0.49 [0.47 - 0.51]
Peng Xue et al. (2020)
0.90 [0.89 - 0.91]
Peng Xue et al. (2020)
0.52 [0.50 - 0.54]
Peng Xue et al. (2020)
0.94 [0.93 - 0.95]
Yu Yao et al. (2020)
0.99 [0.93 - 1.00]
Chunnv Yuan et al. (2020)
0.83 [0.80 - 0.85]
Chunnv Yuan et al. (2020)
0.58 [0.56 - 0.60]
Overall for colposcopy
0.78[0.63 - 0.87]
Q =2426.06, df = 10.00, p = 0.00
I2 = 99.59 [99.52 - 99.66]
0.4 0.6 0.8 1.0
SPECIFICITY

Supplementary Note 1: Search terms and search strategy

Medline (cervical cancer):

1. (("*artificial intelligence*" or "*machine learning*" or "*deep learning*" or "*neural network*") not (decision tree or random forest or nearest neighbo$ or naive bayes or "*vector machine")).ab,hw,kf,ti,nm.
2. diagnosis.ab,hw,kf,ti,nm.
3. "screen*".ab,hw,kf,ti,nm.
4. 2 or 3
5. performance.ab,hw,kf,ti,nm.
6. sensitivity.ab,hw,kf,ti,nm.
7. specificity.ab,hw,kf,ti,nm.
8. accuracy.ab,hw,kf,ti,nm.
9. area under the curve.ab,hw,kf,ti,nm.
10. auc.ab,hw,kf,ti,nm.
11. "calibrat*".ab,hw,kf,ti,nm.
12. 5 or 6 or 7 or 8 or 9 or 10 or 11
13. "cervical cancer".ab,hw,kf,ti,tw.
14. "uterine cervix tumor".ab,hw,kf,ti,tw.
15. "uterine cervical neoplas* ".ab,hw,kf,ti,tw.
16. "*cervical intraepithelial neoplas* ".ab,hw,kf,ti,tw.
17. "uterine cervical cancer* ".ab,hw,kf,ti,tw.
18. "cervical neoplas* ".ab,hw,kf,ti,tw.
19. "squamous intraepithelial lesion* ".ab,hw,kf,ti,tw.
20. "squamous intraepithelial neoplas* ".ab,hw,kf,ti,tw.
21. 13 or 14 or 15 or 16 or 17 or 18 or 19 or 20
22. 4 and 21
23. 12 and 22
24. 1 and 23

Embase (cervical cancer)：

#1'artificial intelligence'/exp OR 'machine learning'/exp OR 'deep learning'/exp OR 'artificial neural network'/exp

#2'decision tree'/exp OR 'random forest'/exp OR 'bayesian learning'/exp OR 'support vector machine'/exp

#3'diagnosis'/exp OR 'diagnostic procedure'/exp OR 'screening'/exp

#4'performance'/exp OR 'sensitivity and specificity'/exp OR 'diagnostic accuracy'/exp OR 'area under the curve'/exp

#5'uterine cervix tumor'/exp OR 'cervical cancer'/exp OR 'uterine cervical neoplas*'

#6'cervical intraepithelial neoplas*'

#7'uterine cervix cancer'

#8'cervical neoplasm*'

#9'squamous intraepithelial lesion*'

#10'squamous intraepithelial neoplas*'
#11#5 OR #6 OR #7 OR #8 OR #9 OR #10
#12#3 AND #11
#13#4 AND #12
#14#1 NOT #2
#15#13 AND #14

IEEE (cervical cancer):
10,"(((((((((No Keywords Specified))) AND ((All Metadata:artificial intelligence) OR (All Metadata:machine learning) OR (All Metadata:deep learning) OR (All Metadata:neural network))) NOT ((All Metadata:decision tree) OR (All Metadata:random forest) OR (All Metadata:naive bayes) OR (All Metadata:nearest neighbor) OR (All Metadata:vector machine))) AND ((No Keywords Specified))) AND ((All Metadata:performance) OR (All Metadata:sensitivity and specificity) OR (All Metadata:accuracy) OR (All Metadata:area under the curve))) AND ((No Keywords Specified))) AND ((All Metadata:screening) OR (All Metadata:diagnosis))) AND ((All Metadata:cervical cancer) OR (All Metadata:uterine cervix tumor) OR (All Metadata:uterine cervical neoplas*) OR (All Metadata:cervical intraepithelial neoplas*) OR (All Metadata:uterine cervical cancer*) OR (All Metadata:cervical neoplas*) OR (All Metadata:squamous intraepithelial lesion*) OR (All Metadata:squamous intraepithelial neoplas*))
9,"(((((((((No Keywords Specified))) AND ((All Metadata:artificial intelligence) OR (All Metadata:machine learning) OR (All Metadata:deep learning) OR (All Metadata:neural network))) NOT ((All Metadata:decision tree) OR (All Metadata:random forest) OR (All Metadata:naive bayes) OR (All Metadata:nearest neighbor) OR (All Metadata:vector machine))) AND ((No Keywords Specified))) AND ((All Metadata:performance) OR (All Metadata:sensitivity and specificity) OR (All Metadata:accuracy) OR (All Metadata:area under the curve))) AND ((No Keywords Specified))) AND ((All Metadata:screening) OR (All Metadata:diagnosis))) AND ((All Metadata:cervical cancer) OR (All Metadata:uterine cervix tumor) OR (All Metadata:uterine cervical neoplas*) OR (All Metadata:cervical intraepithelial neoplas*) OR (All Metadata:uterine cervical cancer*) OR (All Metadata:cervical neoplas*) OR (All Metadata:squamous intraepithelial lesion*) OR (All Metadata:squamous intraepithelial neoplas*))
8,"(((((All Metadata:performance) OR (All Metadata:sensitivity and specificity) OR (All Metadata:accuracy) OR (All Metadata:area under the curve))) AND ((No Keywords Specified))) AND ((All Metadata:screening) OR (All Metadata:diagnosis))) AND ((All Metadata:cervical cancer) OR (All Metadata:uterine cervix tumor) OR (All Metadata:uterine cervical neoplas*) OR (All Metadata:cervical intraepithelial neoplas*) OR (All Metadata:uterine cervical cancer*) OR (All Metadata:cervical neoplas*) OR (All Metadata:squamous intraepithelial lesion*) OR (All Metadata:squamous intraepithelial neoplas*))
7,"(((All Metadata:screening) OR (All Metadata:diagnosis))) AND ((All Metadata:cervical cancer) OR (All Metadata:uterine cervix tumor) OR (All

Metadata:uterine cervical neoplas*) OR (All Metadata:cervical intraepithelial neoplas*) OR (All Metadata:uterine cervical cancer*) OR (All Metadata:cervical neoplas*) OR (All Metadata:squamous intraepithelial lesion*) OR (All Metadata:squamous intraepithelial neoplas*))
6,"(((All Metadata:artificial intelligence) OR (All Metadata:machine learning) OR (All Metadata:deep learning) OR (All Metadata:neural network))) NOT ((All Metadata:decision tree) OR (All Metadata:random forest) OR (All Metadata:naive bayes) OR (All Metadata:nearest neighbor) OR (All Metadata:vector machine))
5,"(All Metadata:cervical cancer) OR (All Metadata:uterine cervix tumor) OR (All Metadata:uterine cervical neoplas*) OR (All Metadata:cervical intraepithelial neoplas*) OR (All Metadata:uterine cervical cancer*) OR (All Metadata:cervical neoplas*) OR (All Metadata:squamous intraepithelial lesion*) OR (All Metadata:squamous intraepithelial neoplas*)
4,"(All Metadata:performance) OR (All Metadata:sensitivity and specificity) OR (All Metadata:accuracy) OR (All Metadata:area under the curve)","",1416666,"March 15, 2021"
3,"(All Metadata:screening) OR (All Metadata:diagnosis)
2,"(All Metadata:decision tree) OR (All Metadata:random forest) OR (All Metadata:naive bayes) OR (All Metadata:nearest neighbor) OR (All Metadata:vector machine)
1,"(All Metadata:artificial intelligence) OR (All Metadata:machine learning) OR (All Metadata:deep learning) OR (All Metadata:neural network)
Cochrane (cervical cancer):

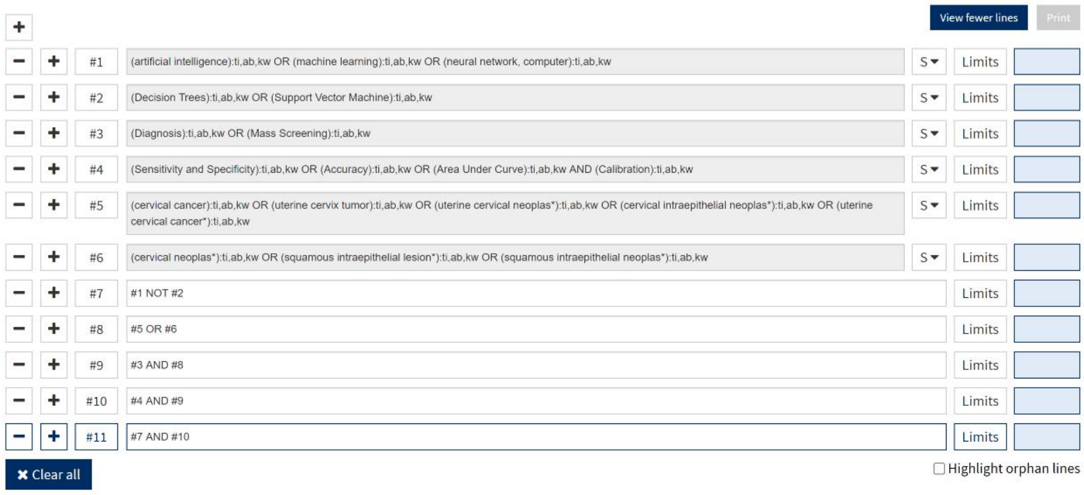

Medline (breast cancer)：

1. (("*artificial intelligence*" or "*machine learning*" or "*deep learning*" or "*neural network*") not (decision tree or random forest or nearest neighbo$ or naive bayes or "*vector machine")).ab,hw,kf,ti,nm.
2. diagnosis.ab,hw,kf,ti,nm.
3. "screen*".ab,hw,kf,ti,nm.
4. 2 or 3

5. performance.ab,hw,kf,ti,nm.
6. sensitivity.ab,hw,kf,ti,nm.
7. specificity.ab,hw,kf,ti,nm.
8. accuracy.ab,hw,kf,ti,nm.
9. area under the curve.ab,hw,kf,ti,nm.
10. auc.ab,hw,kf,ti,nm.
11. "calibrat*".ab,hw,kf,ti,nm.
12. 5 or 6 or 7 or 8 or 9 or 10 or 11
13. breast cancer.ab,hw,kf,ti,tw.
14. 4 and 13
15. 12 and 14
16. 1 and 15

Embase (breast cancer)：

#1'artificial intelligence'/exp OR 'machine learning'/exp OR 'deep learning'/exp OR 'artificial neural network'/exp
#2'decision tree'/exp OR 'random forest'/exp OR 'bayesian learning'/exp OR 'support vector machine'/exp
#3'diagnosis'/exp OR 'diagnostic procedure'/exp OR 'screening'/exp
#4'performance'/exp OR 'sensitivity and specificity'/exp OR 'diagnostic accuracy'/exp OR 'area under the curve'/exp
#5'breast cancer*'/exp
#6 #3 AND #5
#7 #4 AND #6
#8 #1 NOT #2
#9 #7 AND #8

IEEE (breast cancer):
14,((All Metadata:screening) OR (All Metadata:diagnosis))) AND ((All Metadata:breast cancer))) AND ((All Metadata:performance) OR (All Metadata:sensitivity and specificity) OR (All Metadata:accuracy) OR (All Metadata:area under the curve))) AND ((No Keywords Specified))) AND ((All Metadata:artificial intelligence) OR (All Metadata:machine learning) OR (All Metadata:deep learning) OR (All Metadata:neural network))) NOT ((All Metadata:decision tree) OR (All Metadata:random forest) OR (All Metadata:naive bayes) OR (All Metadata:nearest neighbor) OR (All Metadata:vector machine))
13, ((All Metadata:screening) OR (All Metadata:diagnosis))) AND ((All Metadata:breast cancer))) AND ((All Metadata:performance) OR (All Metadata:sensitivity and specificity) OR (All Metadata:accuracy) OR (All Metadata:area under the curve))","",1608,"April 29, 2021"
12,"(((All Metadata:screening) OR (All Metadata:diagnosis))) AND ((All Metadata:breast cancer))
11,"(All Metadata:breast cancer)","",8083,"April 29, 2021"

6,"(((All Metadata:artificial intelligence) OR (All Metadata:machine learning) OR (All Metadata:deep learning) OR (All Metadata:neural network))) NOT ((All Metadata:decision tree) OR (All Metadata:random forest) OR (All Metadata:naive bayes) OR (All Metadata:nearest neighbor) OR (All Metadata:vector machine))
4,"(All Metadata:performance) OR (All Metadata:sensitivity and specificity) OR (All Metadata:accuracy) OR (All Metadata:area under the curve)
3,"(All Metadata:screening) OR (All Metadata:diagnosis)
2,"(All Metadata:decision tree) OR (All Metadata:random forest) OR (All Metadata:naive bayes) OR (All Metadata:nearest neighbor) OR (All Metadata:vector machine)
1,"(All Metadata:artificial intelligence) OR (All Metadata:machine learning) OR (All Metadata:deep learning) OR (All Metadata:neural network)

Cochrane (breast cancer):

#1	(artificial intelligence):ti,ab,kw OR (machine learning):ti,ab,kw OR (neural network, computer):ti,ab,kw	S	Limits
#2	(Decision Tree):ti,ab,kw OR ("support vector machine"):ti,ab,kw	S	Limits
#3	(breast cancer):ti,ab,kw	S	Limits
#4	(diagnosis):ti,ab,kw OR (mass screening):ti,ab,kw	S	Limits
#5	(sensitivity and specificity):ti,ab,kw OR (accuracy):ti,ab,kw OR (area under curve):ti,ab,kw OR (calibration):ti,ab,kw	S	Limits
#6	#1 NOT #2		Limits
#7	#3 AND #4		Limits
#8	#5 AND #7		Limits
#9	#6 AND #8		Limits

Supplementary Table 2 Contingency tables extracted from included studies (73 tables from 20 studies)

First author	Year	Disease	Total	TP	FP	FN	TN	sensitivity	specificity	AUROC
Adachi et al.	2020	breast cancer	85	48	5	4	28	92.60%	82.80%	
Adachi et al.	2020	breast cancer	85	44	5	8	28	84.70%	84.10%	
Bao et al.	2020	cervical cancer	2076	643	414	151	868	81.00%	67.70%	
Bao et al.	2020	cervical cancer	69906	980	3579	108	65239	90.10%	94.80%	
Bao et al.	2020	cervical cancer	68906	917	2303	171	65515	84.30%	95.20%	
Cho et al.	2020	cervical cancer	117	54	11	29	23	65.70%	67.90%	0.708
Cho et al.	2020	cervical cancer	117	52	12	31	22	62.90%	63.50%	0.685
Cho et al.	2020	cervical cancer	116	55	10	28	23	66.70%	70.60%	0.781
Cho et al.	2020	cervical cancer	116	55	10	28	23	66.70%	69.90%	0.739
Holmström et al.	2021	cervical cancer	361	16	48	3	294	84.20%	86.00%	
Holmström et al.	2021	cervical cancer	361	24	5	4	328	85.70%	98.50%	
Holmström et al.	2021	cervical cancer	361	45	48	2	266	95.70%	84.70%	
Holmström et al.	2021	cervical cancer	361	19	74	0	268	100.00%	78.40%	
Holmström et al.	2021	cervical cancer	361	5	24	0	332	100.00%	93.30%	
Holmström et al.	2021	cervical cancer	361	3	61	11	286	21.40%	82.40%	
Hu et al.	2019	cervical cancer	8917	212	1480	16	7209	92.98%	83.90%	
Hunt et al.	2021	cervical cancer	616	166	178	13	259	93.00%	59.00%	
Hunt et al.	2021	cervical cancer	616	172	169	7	268	96.00%	61.00%	0.860
Kim et al.	2020	breast cancer	320	142	29	18	131	88.75%	81.87%	0.940
Kim et al.	2020	breast cancer	654	200	101	18	335	91.70%	76.80%	0.938
Kim et al.	2020	breast cancer	750	234	99	16	401	93.60%	80.20%	0.953
Kim et al.	2020	breast cancer	1858	559	103	60	1136	90.30%	91.70%	0.970
Kim et al.	2020	breast cancer	3262	993	303	94	1872	91.40%	86.00%	0.959

Kim et al.	2020	breast cancer	320	120	45	40	115	75.27%	71.96%	0.810
Qi et al.	2018	breast cancer	1359	854	75	59	371	93.54%	83.18%	0.928
Qi et al.	2018	breast cancer	1359	852	105	61	341	93.31%	76.45%	0.936
Qi et al.	2018	breast cancer	1359	842	101	71	345	92.22%	77.35%	0.928
Qi et al.	2018	breast cancer	1359	440	55	20	844	95.65%	93.88%	0.982
Qi et al.	2018	breast cancer	1354	426	44	34	850	92.61%	94.55%	0.979
Qi et al.	2018	breast cancer	1359	402	30	58	869	87.39%	96.66%	0.980
Salim et al.	2020	breast cancer	113663	498	3728	241	109196	67.40%	96.70%	
Salim et al.	2020	breast cancer	113663	495	3839	244	109085	67.00%	96.60%	
Salim et al.	2020	breast cancer	113663	605	3839	134	109085	81.90%	96.60%	
Salim et al.	2020	breast cancer	113663	572	3839	167	109085	77.40%	96.60%	
Salim et al.	2020	breast cancer	114263	592	3762	147	109762	80.10%	97.20%	
Sasaki et al.	2020	breast cancer	620	60	181	10	369	85.71%	67.09%	
Sasaki et al.	2020	breast cancer	620	65	302	5	248	92.86%	45.09%	
Sasaki et al.	2020	breast cancer	620	37	4	33	546	52.86%	99.27%	
Sasaki et al.	2020	breast cancer	620	65	302	5	248	92.86%	45.09%	
Sasaki et al.	2020	breast cancer	620	62	77	8	473	89.00%	86.00%	
Sasaki et al.	2020	breast cancer	620	62	77	8	473	89.00%	86.00%	
Sasaki et al.	2020	breast cancer	177	20	41	6	110	77.00%	73.00%	
Sasaki et al.	2020	breast cancer	133	41	23	1	68	98.00%	75.00%	
Suh et al.	2020	breast cancer	301	50	21	4	226	88.30%	87.90%	0.954
Suh et al.	2020	breast cancer	301	47	29	7	218	87.00%	88.40%	0.952
Tanaka et al.	2019	breast cancer	850	397	68	51	334	88.60%	83.10%	0.938
Tanaka et al.	2019	breast cancer	850	392	76	56	326	87.50%	81.10%	0.919
Tanaka et al.	2019	breast cancer	850	403	71	45	331	90.00%	82.30%	0.926

Tanaka et al.	2019	breast cancer	154	70	10	7	67	90.90%	87.00%	0.951
Tanaka et al.	2019	breast cancer	154	67	12	10	65	87.00%	84.40%	0.945
Tanaka et al.	2019	breast cancer	154	71	12	6	65	92.20%	84.40%	0.935
Wentzensen et al.	2021	cervical cancer	409	46	194	7	162	87.00%	45.60%	0.740
Wentzensen et al.	2021	cervical cancer	409	46	212	7	144	87.00%	40.50%	
Xiao et al.	2020	breast cancer	216	112	29	28	47	80.00%	61.84%	0.710
Xiao et al.	2020	breast cancer	235	75	25	5	130	93.75%	83.87%	0.890
Xiao et al.	2020	breast cancer	235	76	52	4	103	95.00%	66.45%	0.810
Xiao et al.	2020	breast cancer	216	136	30	4	46	97.14%	60.53%	0.790
Xue et al.	2020	cervical cancer	3887	1074	145	420	2248	71.90%	93.90%	
Xue et al.	2020	cervical cancer	3887	1352	1153	142	1240	90.50%	51.80%	
Xue et al.	2020	cervical cancer	3887	983	240	511	2153	65.80%	90.00%	
Xue et al.	2020	cervical cancer	3887	1304	1224	190	1169	87.30%	48.90%	
Xue et al.	2020	cervical cancer	4134	1247	1148	247	1492	83.50%	52.00%	
Xue et al.	2020	cervical cancer	3887	903	123	591	2270	60.40%	94.90%	
Yu et al.	2020	cervical cancer	135	51	1	2	81	95.68%	98.72%	
Yuan et al.	2020	cervical cancer	5384	1474	1412	535	1963	73.37%	58.16%	
Yuan et al.	2020	cervical cancer	2233	1022	180	175	856	85.38%	82.63%	
Zhang et al.	2021	breast cancer	210	76	18	17	99	81.70%	84.60%	0.900
Zhang et al.	2021	breast cancer	707	408	34	39	226	91.30%	86.90%	0.960
Zhang et al.	2021	breast cancer	707	441	96	6	164	98.70%	63.10%	
Zhou et al.	2019	breast cancer	307	187	31	19	70	90.80%	69.30%	0.859
Zhou et al.	2019	breast cancer	307	189	39	17	62	91.80%	61.40%	0.856
Zhou et al.	2019	breast cancer	307	178	30	28	71	86.40%	70.30%	0.858
Zhou et al.	2019	breast cancer	307	203	41	3	60	98.50%	59.40%	
